# The CHIRPY DRAGON intervention in preventing obesity in Chinese primary-school--aged children: A cluster-randomised controlled trial

**DOI:** 10.1371/journal.pmed.1002971

**Published:** 2019-11-26

**Authors:** Bai Li, Miranda Pallan, Wei Jia Liu, Karla Hemming, Emma Frew, Rong Lin, Wei Liu, James Martin, Mandana Zanganeh, Kiya Hurley, Kar Keung Cheng, Peymane Adab

**Affiliations:** 1 Centre for Exercise, Nutrition and Health Sciences, University of Bristol, Beacon House, Queens Road, Bristol, United Kingdom; 2 Institute of Applied Health Research, University of Birmingham, Edgbaston, Birmingham, United Kingdom; 3 School Health Unit, Guangzhou Centre of Disease Control and Prevention, Guangzhou, P.R.China; Harvard Medical School, UNITED STATES

## Abstract

**Background:**

In countries undergoing rapid economic transition such as China, rates of increase in childhood obesity exceed that in the West. However, prevention trials in these countries are inadequate in both quantity and methodological quality. In high-income countries, recent reviews have demonstrated that school-based prevention interventions are moderately effective but have some methodological limitations. To address these issues, this study evaluated clinical- and cost- effectiveness of the Chinese Primary School Children Physical Activity and Dietary Behaviour Changes Intervention (CHIRPY DRAGON) developed using the United Kingdom Medical Research Council complex intervention framework to prevent obesity in Chinese primary-school–aged children.

**Methods and findings:**

In this cluster-randomised controlled trial, we recruited 40 state-funded primary schools from urban districts of Guangzhou, China. A total of 1,641 year-one children with parent/guardian consent took part in baseline assessments prior to stratified randomisation of schools (intervention arm, 20 schools, *n* = 832, mean age = 6.15 years, 55.6% boys; control arm *n* = 809, mean age = 6.14 years, 53.3% boys). The 12-month intervention programme included 4 school- and family-based components delivered by 5 dedicated project staff. We promoted physical activity and healthy eating behaviours through educational and practical workshops, family activities, and supporting the school to improve physical activity and food provision. The primary outcome, assessed blind to allocation, was between-arm difference in body mass index (BMI) z score at completion of the intervention. A range of prespecified, secondary anthropometric, behavioural, and psychosocial outcomes were also measured. We estimated cost effectiveness based on quality-adjusted life years (QALYs), taking a public sector perspective. Attrition was low with 55 children lost to follow up (3.4%) and no school dropout. Implementation adherence was high. Using intention to treat analysis, the mean difference (MD) in BMI z scores (intervention − control) was −0.13 (−0.26 to 0.00, *p* = 0.048), with the effect being greater in girls (MD = −0.18, −0.32 to −0.05, *p* = 0.007, *p* for interaction = 0.015) and in children with overweight or obesity at baseline (MD = −0.49, −0.73 to −0.25, *p* < 0.001, *p* for interaction < 0.001). Significant beneficial intervention effects were also observed on consumption of fruit and vegetables, sugar-sweetened beverages and unhealthy snacks, screen-based sedentary behaviour, and physical activity in the intervention group. Cost effectiveness was estimated at £1,760 per QALY, with the probability of the intervention being cost effective compared with usual care being at least 95% at a willingness to pay threshold of £20,000 to 30,000 per QALY. There was no evidence of adverse effects or harms. The main limitations of this study were the use of dietary assessment tools not yet validated for Chinese children and the use of the UK value set to estimate QALYS.

**Conclusions:**

This school- and family-based obesity prevention programme was effective and highly cost effective in reducing BMI z scores in primary-school–aged children in China. Future research should identify strategies to enhance beneficial effects among boys and investigate the transferability of the intervention to other provinces in China and countries that share the same language and cultures.

**Trial registration:**

ISRCTN Identifier ISRCTN11867516.

## Abstract 2

Bai Li and colleagues reveal a healthy eating and activity intervention in Chinese children that reduces obesity

## Author summary

**Why was this study done?:**

- In low- and middle-income countries (LMIC) where childhood obesity is increasing rapidly, there is a lack of rigorous development and evaluation of prevention interventions.
- Previous childhood obesity prevention trials conducted in China have not provided robust, high-quality evidence of effective interventions.
- Without effective prevention programs, China is estimated to have 50 million children with overweight or obesity by 2030.

**What did the researchers do and find?:**

- Guided by the United Kingdom Medical Research Council’s complex intervention framework, we developed a school- and family-based intervention (the Chinese Primary School Children Physical Activity and Dietary Behaviour Changes Intervention [CHIRPY DRAGON] programme) for preventing obesity in urban, primary-school–aged children in China.
- A total of 40 primary schools (1,461 children aged 6 years) were recruited from Guangzhou, China. The children took part in weight status and behavioural assessments before their schools were randomly divided into 2 groups. One group of schools received a package of activities designed to increase healthy eating and physical activity (the CHIRPY DRAGON program) over 12 months, and the other group of schools continued with their usual activities. After 12 months, all children taking part were assessed again, and these measures were compared across the 2 school groups. We also collected and analysed comprehensive data on the costs of the CHIRPY DRAGON programme and how it was implemented.
- We found that the CHIRPY DRAGON programme was mainly delivered as planned. Children, particularly girls, in the group of schools receiving the program had healthier weight status, dietary intake, and physical activity levels at the end of the 12-month period compared with children in the schools not receiving the programme. The programme was also good value for money.

**What do these findings mean?:**

- This school- and family-based programme may be an effective and cost-effective intervention for the prevention of obesity in children in urban China.
- Future research should identify strategies to enhance beneficial effects of the programme among boys.
- It is not yet determined whether this intervention programme can be successfully transferred to other contexts, but it may be effective in other locations that share similar characteristics to urban China.

## Introduction

Childhood obesity is recognised by the World Health Organisation (WHO) as one of the most serious but preventable global public health challenges [1]. In China, the combined prevalence of overweight and obesity has increased more rapidly and over a shorter time period than other countries [2]. The prevalence of being overweight or obese in school children increased from around 1% (in both genders) in 1985 to 28.2% in boys and 16.4% in girls in 2015 [2]. Without effective interventions, China is estimated to have 50 million children with overweight or obesity by 2030 [2]. In order to achieve the grand vision set out in the Government’s Healthy China 2030 policy document [3], there is an urgent need for effective preventive interventions to address this rapid increase.

However, in the most recent systematic review of Chinese childhood obesity intervention trials, most studies are treatment focused and used a nonrandomised design. None were rated as high-quality methodologically, mainly because of selection bias and lack of blinded assessments, report of dropouts, sufficient adjustment of confounders, and intention to treat analysis. The authors of the review highlighted the need for higher quality studies to evaluate multicomponent preventive interventions targeting obesity in children in China [4]. Internationally, very few intervention studies have been conducted in low- and middle-income countries (LMIC) [5].

To address these gaps, we developed (2009–2015) [6,7] and evaluated (2015–2017) an evidence-based, multicomponent intervention aiming to prevent obesity in Chinese primary school children in the Chinese Primary School Children Physical Activity and Dietary Behaviour Changes Intervention (CHIRPY DRAGON) study, using the guidelines from the United Kingdom Medical Research Council (MRC) framework for complex interventions [8]. We report the effectiveness and cost effectiveness of the intervention programme here.

## Methods

### Study design and participants

We conducted a parallel, two-arm, cluster-randomised controlled trial of the CHIRPY DRAGON intervention in 40 primary schools in Guangzhou, the largest and one of the most socioeconomically advanced cities in South China. We obtained ethics approvals from the Life and Health Sciences Ethical Review Committee at the University of Birmingham, UK (2 March 2015) and the Ethical Committee of Guangzhou Centre for Disease Control and Prevention, China (1 December 2014). The full trial protocol has been published and is available online [9]. We implemented the protocol without changes.

All nonboarding, state-funded primary schools (clusters) in traditional urban districts of Guangzhou were eligible (*n* = 353). A research team member (WL) used a random number generator to select 40 schools, which were invited to take part in the trial. Through support from local education and health authorities (an official support letter was sent to each of the sampled schools) and personal visits (with written information sheet and consent form) or telephone communication from the research team members, all 40 schools agreed to take part. Using a random number generator, a research team member selected 1 year-one class from each school to participate in study measurements (average number of classes per year is 4; range: 2 to 8). We invited all children in these classes to take part with active consent sought from their parents or guardians. However, for equity and practical reasons, we delivered child-focused, school-based intervention components to all classes of year one. The school meal intervention component that targeted school lunch providers (described in S1 Table) was likely to impact on the entire school, because the catering team provided the same menu to children across all years in each school.

### The intervention and its development

Children in the intervention schools received the CHIRPY DRAGON programme, described in detail in the published trial protocol [9] and summarised in S1 Table. Briefly, the developed intervention programme included 4 school- and family-based components targeting children, main carers (parents or guardians and grandparents) as well as school physical activity and food provision to encourage physical activity and healthy eating behaviours in children both within and outside of school. A range of stakeholders (parents, grandparents, school teachers, managers and workers of school catering providers, and managers of food stores located near schools) contributed to the prioritisation of intervention components that had previously been identified as promising from published international and Chinese systematic reviews and our own formative research [6,7]. We incorporated a range of behaviour-change techniques and social marketing principles in designing the intervention and tested and refined the programme through a feasibility study [6].

### Intervention delivery

We employed and trained 5 full-time Chinese project staff (known as CHIRPY DRAGON teachers) to deliver the intervention over 12 months. Each of the 5 CHIRPY DRAGON teachers was responsible for the coordination and delivery of the intervention activities in 4 intervention schools. We gave contact information of the study management team and CHIRPY DRAGON teachers to families and school staff and advised them to make contact if they had any concerns or queries. We provided school principals and class teachers with a programme handbook that explained all intervention activities and the support for intervention delivery that was required from the school staff. Further information on the staff training programme and quality control of intervention delivery is provided in Appendix 1.

### Comparator

Schools assigned to the control arm continued with their usual provision during the full trial period with no access to any of the CHIRPY DRAGON intervention activities and resources.

### Participant assessment

We undertook baseline assessments when participating children were in year one (age 6 to 7 years), in the first/autumn term (September to December 2015). We started delivering the 12-month intervention programme in the second/spring term, following a school winter holiday with follow-up measurements undertaken at the end of the intervention period (April to July 2017), when the children were in year two (age 7 to 8 years).

### Randomisation and masking

Randomisation took place after baseline measurements were obtained from participating children (January 2016). A trial statistician (JM) allocated schools to the intervention and control groups using a computer generated sequence (ralloc function in Stata Statistical Software, Release 14, StataCorp LP, https://www.stata.com), stratified on 2 school-level factors: school provision of midmorning snacks and availability of indoor activity space. We selected these 2 factors in consultation with the lead trial statistician (KH) and the local education authority with knowledge of key factors that could potentially influence dietary and physical activity behaviours of children in local primary schools.

Because of the nature of the intervention, school staff, children, family members, and project staff who delivered the intervention could not be masked to group allocation during the intervention period. However, we employed independent, masked, trained assessors who were not involved in any part of the intervention delivery to undertake all outcome measurements. Risk of bias at each stage of the trial is summarised in a timeline cluster diagram [10] (S1 Appendix).

### Outcomes and data collection methods

The primary outcome for clinical effectiveness as specified in the trial protocol was the difference in body mass index (BMI) standard deviation scores (z scores) between arms at completion of the 12-month intervention. Prespecified secondary outcomes (Table 1) included the proportion of children with overweight or obesity; waist circumference; body fat percentage; consumption of fruit and vegetables, unhealthy snacks and sugar-sweetened beverages; time spent in moderate to vigorous physical activity (MVPA); sedentary behaviours; and blood pressure. To consider the wider psychosocial effects of the intervention (potential benefits and harms), we also assessed health-related quality of life and social acceptance.

**Table 1:** Summary of the secondary outcomes measures for the CHIRPY DRAGON trial and method of measurement.

Secondary outcomes	Assessment methods or instruments
**Anthropometric measures:**	
Overweight/obesity	Standing height: TGZ-type height tester (Dalian); Weight: an electronic scale (JH-1993T, weighing Apparatus Co. Ltd., Dalian, Dalian, China); Overweight/obesity: defined by WHO 2007 BMI Growth Charts: above +2 z scores [11]
Body fat %	Single-frequency ImpediMed machine
Waist circumference	A nonstretch tape measure
**Eating behaviours**	
Daily average portions of fruit and vegetables	Derived from adapted S [12]
Proportion of children consuming ≥5 portions of fruit and vegetables daily	Derived from adapted SFFQ from University of Leeds [12]
Weekly average servings of unhealthy snacks and sugar-added drinks	Derived from adapted SFFQ from University of Leeds [12]
**Physical activity**	
Objectively measured time spent in MVPA (minutes/24 hours)	GENEActiv Original, Activinsights Ltd, Cambridge
Parent-reported time spent in MVPA (minutes/24 hours)	Adapted Godin Leisure-Time Exercise Questionnaire [13]
Proportion of children achieving ≥60 minutes MVPA/24 hours (objectively measured)	GENEActiv Original, Activinsights Ltd, Cambridge
Proportion of children achieving ≥60 minutes MVPA/24 hours (parent reported)	Adapted Godin Leisure-Time Exercise Questionnaire [13]
Proportion of children engaging in active sports, dance, or games for at least once in the weekend (self-reported)	Purposely designed questionnaire
**Sedentary behaviours**	
Objectively measured sedentary behaviours (minutes/24 hours)	GENEActiv Original, Activinsights Ltd, Cambridge
Parent-reported sedentary behaviours (minutes/24 hours)	Adapted Godin Leisure-Time Exercise Questionnaire [13]
Proportion of children engaging in screen viewing behaviour in the week days (self-reported)	Purposely designed questionnaire
Proportion of children engaging in screen viewing behaviour at weekends (self-reported)	Purposely designed questionnaire
**Psychosocial outcomes (potential benefits and harms)**	
Self-reported health-related quality of life	Validated Chinese version of PedsQL 4.0 [14] and The Child Health Utility 9D (CHU 9D) [15]
Social acceptance	Translated version of the social acceptance domain from the Kidscreen-52 health questionnaire [16]
**Blood pressure**	
Systolic blood pressure	Validated, automated monitors, Omron HEM-7211, Dalian
Diastolic blood pressure	Validated, automated monitors, Omron HEM-7211, Dalian

**Abbreviations:** CHIRPY DRAGON, Chinese Primary School Children Physical Activity and Dietary Behaviour Changes Intervention; MVPA, moderate to vigorous physical activity; SFFQ, short food frequency questionnaire

We have reported data collection methods in the published trial protocol [9] and provided a summary of this (S2 Table). All measurements were undertaken by trained researchers, masked to allocation, using standard protocols. We used data on height and weight to calculate BMI z score, based on WHO 2007 growth charts [11]. We used validated objective (GENEActiv Original accelerometers, Activinsights Ltd, Cambridge) and parent-reported methods to assess children’s physical activity and sedentary behaviours. To extract the data from the Geneactiv devices, we used a bulk import Microsoft Excel macro-enabled spreadsheet provided by the device developers (Available at: https://open.geneactiv.org/geneactiv_macros.html). Activity levels were assigned as sedentary, light, moderate, or vigorous using previously published child-specific thresholds [17]. We defined a valid day's wear as more than 10 hours wear time in a 24-hour period [18] and set pragmatic limits for valid activity levels (i.e., no more than 16 hours spent in either sleep, sedentary or light activity and no more than 6 hours total in moderate and/or vigorous activity). We excluded all data exceeding these limits from the analyses and only included children with a minimum of 2 valid week days and 1 valid weekend day recorded.

### Process evaluation

The detailed methods and findings of the process evaluation will be published separately but are briefly summarised in this paper. Guided by the UK MRC process evaluation recommendations [19], we used various methods to collect quantitative and qualitative data on intervention implementation, contextual factors, and mechanisms of impacts. Data collection methods included implementation record forms (completed by the CHIRPY DRAGON teachers), observations by nondelivery staff, child daily challenge self-monitoring cards (including written parental feedback), and focus groups/interviews with study participants (including parents, grandparents, school staff, children, and managers and workers of school catering companies/units).

### Economic evaluation

We conducted a cost-effectiveness analysis (CEA) based on the BMI z scores and a cost-utility analysis (CUA), using quality-adjusted life years (QALYs), calculated from the Child Health Utility 9D (CHU-9D-CHN) [20] measure of health-related quality of life [15] using the UK value set. All costs and outcomes were assessed at 12 months. To estimate costs, resource use data was collected alongside the trial. All costs are reported in Chinese Yuan at 2016 to 2017 prices and converted into Pounds/United States dollars using Gross Domestic Product Purchasing Power Parities (GDP PPPs) [21]. For the main economic analysis, all costs associated with the delivery of the intervention were included, comprising of CHIRPY DRAGON teachers’ and workshop assistants’ time; transport; incentives; all intervention materials; telephone costs; and office stationary. To estimate cost effectiveness, we calculated the incremental cost-effectiveness ratio (ICER) based on the fully adjusted costs and effects. In the absence of an agreed Chinese threshold for the value of a QALY, decision uncertainty was assessed using established UK and US thresholds [22,23] and presented using Cost Effectiveness Acceptability Curves (CEAC).

### Sample size

We calculated that 1,640 children needed to be recruited from 40 schools to have 80% power at a 5% significance level to detect a difference of 0.17 units in the mean BMI z scores between arms, assuming an average of 45 children per cluster, BMI z score SD of 1.39, an intraclass correlation coefficient of 0.01 (values were from a cross-sectional study in the same setting [24]), loss to follow up of 10%, and a correlation between baseline and follow up observations of 0.70.

### Statistical analysis

We assessed baseline characteristics by study arm. We used a mixed-effect linear model to analyse the primary outcome (mean difference [MD] in BMI z scores between the 2 arms post intervention). We used logistic or linear link functions to analyse binary or continuous secondary outcomes. For continuous outcomes, we checked the distribution of residuals for normality. For all outcomes, the residuals were normally distributed and no transformations were used. In primary analyses, we adjusted for clustering (with a random effect) and baseline value of the outcome only. In secondary analyses, we additionally adjusted for prespecified school- and child-level covariates. These included factors used in randomisation and important sociodemographic (i.e., sex and mother’s education level) and health behaviour factors (consumptions of fruit/vegetables and unhealthy snacks/sugar-added drink, and MVPA and sedentary minutes per day).

As prespecified in the trial protocol, we used interaction terms to examine whether any difference in treatment effects varied by child’s sex, mother’s education level (university/higher education versus nonuniversity/lower education), and child weight status at baseline (overweight/obese versus non-overweight/obese). Given the very high retention rate and a high level of data completeness for the primary outcome and most secondary outcomes, we did not account for missing data. We conducted all analyses as intention to treat on randomised participants with available data in STATA version 13.

This trial is prospectively registered with International Standard Randomised Controlled Trials, number ISRCTN11867516.

## Results

**Fig 1:**
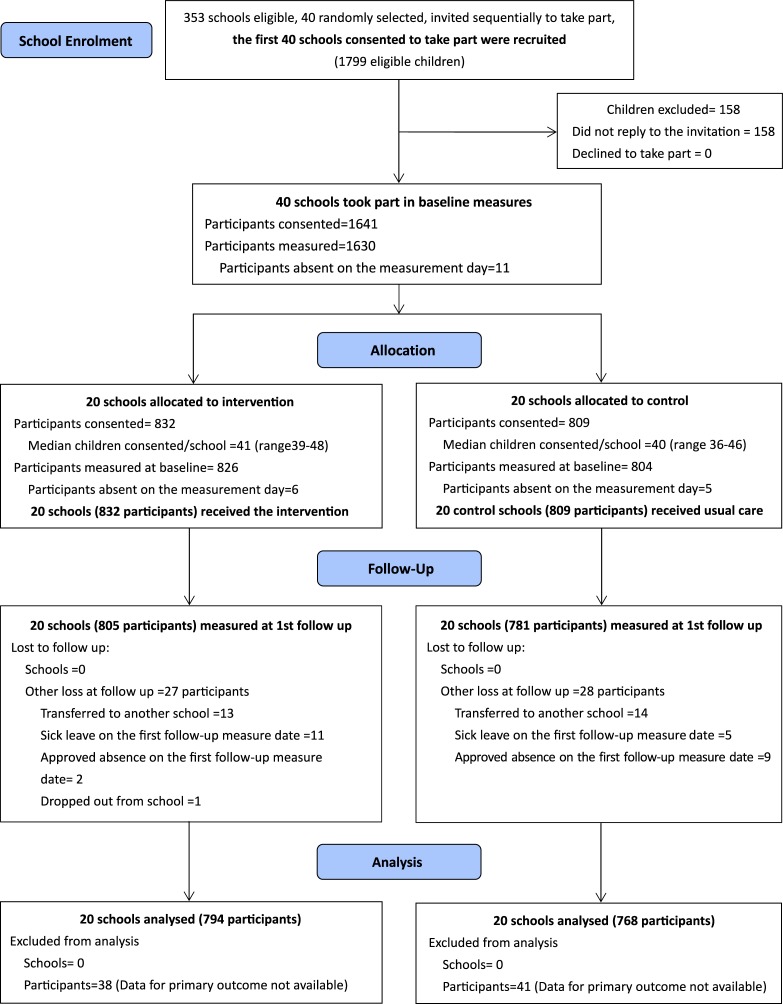
Participants flow chart of the CHIRPY DRAGON trial. CHRIPY DRAGON, Chinese Primary School Children Physical Activity and Dietary Behaviour Changes Intervention.

Within the 40 participating schools, 1,799 children were eligible (September 2015). A total of 158 did not reply to our recruitment letters. The remaining 1,641 (91.2%) consented and participated in study measurements (Fig 1). We randomly allocated 20 schools (832 consented children) to the intervention programme and 20 schools (809 consented children) to usual care. Baseline characteristics of the study participants were well balanced between the 2 groups (Table 2). Loss to follow up was lower (3.4% overall, 3.3% in the intervention, and 3.5% in the control arms) than the estimated level used (10%) in the sample size determination. No schools dropped out of the trial. Missing and/or invalid data for the primary outcome was very low at both measurement points. We included 794 children (95.4%) from the intervention group and 768 (95.0%) from the control group in the primary outcome analysis. Over half of the children contributed valid data on objectively measured MVPA/sedentary time (56.6%) and body fat% (54.0%) at both measurement points. We successfully collected data on 85.9% to 96.8% of children for all other secondary outcomes. We found no differences between the 2 study groups in completeness of outcome measures. Comparison between trial completers (those who completed assessments at the primary follow-up, *n* = 1,586) and those lost to follow up (*n* = 55) showed no major differences in child baseline characteristics (S3 Table).

**Table 2:** Baseline characteristics of children participating in the CHIRPY DRAGON study by trial arm.

Characteristics	Intervention group (20 schools) *N* = 832	Control group (20 schools) *N* = 809
**Age (years)**	6.15 (0.36)	6.14 (0.35)
**Sex**		
Boys	463 (55.6%)	431 (53.3%)
Girls	369 (44.4%)	378 (46.7%)
**Mother’s highest education level**		
** Lower education**		
None	1 (0.1%)	1 (0.1%)
School education (Primary and Middle schools)	167 (20.5%)	137 (17.8%)
Occupation college	160 (19.6%)	132 (17.2%)
** Higher education**		
University education (Undergraduate level)	434 (53.3%)	433 (56.3%)
Postgraduate education	53 (6.5%)	66 (8.6%)
**BMI z- score**	-0.13 (1.30)	-0.13 (1.30)
**Weight (kg)**	22.30 (4.32)	22.19 (4.28)
**Height (cm)**	119.77 (5.47)	119.49 (5.50)
**Waist circumference**	53.71 (5.79)	53.71 (5.76)
**Body fat %**	21.30 (6.23)	21.53 (6.05)
Weight status*		
Underweight	37 (4.5%)	44 (5.5%)
Healthy weight	637 (77.5%)	610 (76.6%)
Overweight	92 (11.2%)	83 (10.5%)
Obese	56 (6.8%)	59 (7.4%)
**Systolic blood pressure (mmHg)**	101.99 (9.24)	101.29 (9.20)
**Diastolic blood pressure (mmHg)**	61.69 (7.17)	61.05 (7.14)
**≥5 portions of fruit and vegetable consumed per day**		
Yes	82 (10.4%)	66(8.9%)
No	705 (89.6%)	675(91.1%)
**Daily average servings of fruit and vegetables, median[IQR]**	3.00 [2.00–4.00]	3.00 [2.00–4.00]
**Weekly average servings of unhealthy snacks and sugar-added drinks~, median [IQR]**	2.50 [0.00–4.50]	2.00 [0.00–3.50]
**Objectively measured time in MVPA (minutes/24hours)**	64.7 (30.8)	67.9 (29.1)
**Parent-reported time in MVPA (minutes/24hours),median [IQR]**	120.0 [77.1–165.7]	115.7 [68.6–167.1]
**≥ 60 mins MVPA/24 hours (objectively measured)**		
Yes	304 (50.3%)	299 (56.1%)
No	301 (49.8%)	234 (43.9%)
**≥ 60 mins MVPA/24 hours (parent reported)**		
Yes	693 (84.9%)	647 (83.1%)
No	123 (15.1%)	132 (16.9%)
**Child engaged in active sports/dancing/games at least once in the last weekend**		
Yes	514 (62.8%)	490 (61.6%)
No	304 (37.2%)	306 (38.4%)
**Objectively measured sedentary time (minutes/24 hours)**	440.3 (90.1)	442.8 (87.0)
**Parent-reported sedentary time (minutes/24 hours)**	199.4 (145.7)	202.2 (146.7)
**Time spent on screen-based sedentary behaviour during weekdays**		
0 hour	276 (33.8%)	238 (29.9%)
Within 30 minutes	337 (41.2%)	336 (42.2%)
0.5–1 hour	109 (13.3%)	103 (12.9%)
1–2 hours	62 (7.6%)	70 (8.8%)
2–3 hours	14 (1.7%)	24 (3.0%)
3 or more hours	19 (2.3%)	25 (3.1%)
**Time spent on screen-based sedentary behaviour on weekend days**		
0 hour	169 (20.7%)	157 (19.7%)
Within 30 minutes	307 (37.5%)	322 (40.5%)
0.5–1 hour	153 (18.7%)	126 (15.8%)
1–2 hours	97 (11.9%)	88 (11.1%)
2–3 hours	40 (4.9%)	45 (5.7%)
3 or more hours	52 (6.4%)	58 (7.3%)

Data are mean (SD) or n (%), unless specified as median [IQR].

*based on WHO 2007 Growth Chart.

~Unhealthy snack consumption is estimated as the sum of average servings of salty high fat snacks (e.g. crisp, deep fried snacks), sweet high fat snacks (e.g., chocolates, cake, ice cream, and biscuits), candies and sugared beverages (e.g., carbonated drinks) in the previous week.

**Abbreviations:** BMI, body mass index; CHIRPY DRAGON, Chinese Primary School Children Physical Activity and Dietary Behaviour Changes Intervention; IQR, interquartile range; MVPA, moderate to vigorous physical activity

We did not receive any reports of adverse events related to the intervention. The odds ratio (OR) for children becoming underweight in the intervention arm compared with the comparator arm was 0.94 (0.46–1.94, *p* = 0.865) and 0.73 (0.34–1.57, *p* = 0.416) in the baseline-adjusted and further-adjusted models, respectively, suggesting no statistically significant difference between arms in the risk of becoming underweight.

### Primary outcome

At 12 months (end of intervention period), the mean BMI z score was significantly lower in the intervention compared with the control group, MD = −0.13, 95% CI: −0.26 to 0.00, *p* = 0.048 in the baseline-adjusted model; MD = −0.13, 95% CI: −0.26 to −0.01, *p* = 0.041 in the further-adjusted model (Table 3).

**Table 3:** Adjusted differences for the primary (BMI z score) and secondary outcomes between intervention and control groups at 12 months.

Characteristics	Intervention group		Control group		MD or OR (95% CI), P value	
	*N*^a^	Mean (SD)/Median [IQR] / %	*N*^a^	Mean (SD)/Median [IQR] /%	Baseline adjusted^b^ (primary analysis)	Further adjusted^c^ (secondary analysis)
**Adiposity-related outcomes**						
**BMI z score**^**d**^	804	-0.35 (1.22)	777	-0.23 (1.34)	-0.13 (-0.26 to 0.00), p = 0.048	-0.13 (-0.26 to -0.01), p = 0.041
**Obese/Overweight**^**e**^						
No	679	84.5%	631	81.2%	NA	NA
Yes	125	15.5%	146	18.8%	0.53 (0.27 to 1.05), p = 0.067	0.65 (0.31 to 1.36), p = 0.258
**Waist circumference**	805	57.45 (6.82)	781	57.85 (6.87)	-0.37 (-0.85 to 0.11), p = 0.128	-0.53 (-1.06 to -0.01), p = 0.047
**Body fat %**^**f**^	476	18.96 (5.64)	431	19.95 (5.64)	-0.01 (-0.03 to 0.01), p = 0.171	-0.01 (-0.03 to 0.00), p = 0.136
**Blood pressure**						
Systolic blood pressure (mmHg)	805	101.54 (8.68)	781	101.33 (8.99)	-0.04 (-1.37 to 1.29), p = 0.953	-0.24 (-1.58 to 1.10), p = 0.723
Diastolic blood pressure (mmHg)	805	60.82 (7.15)	781	61.22 (7.18)	-0.65 (-1.97 to 0.67), p = 0.332	-0.71 (-2.01 to 0.59), p = 0.287
**Behavioural outcomes**						
**Daily average servings of fruit and vegetables**	787	3·00 [2·00–4·00]	741	3·00 [2·00–4·00]	0.33 (0.14 to 0.52), p = 0.001	0.34 (0.17 to 0.51), p<0.001
**≥5 portions of fruit and vegetables**^**g**^						
No	653	82.1%	696	90.5%	NA	NA
Yes	142	17.9%	73	9.5%	2.00 (1.45 to 2.76), p<0.001	2.12 (1.47 to 3.07), p<0.001
**Weekly average servings of unhealthy snacks and sugar-added drinks**	770	1.00 [0.00–3.00]	732	2.50 [0.00–3.50]	-0.81(-1.42 to -0.20), p = 0.010	-0.76 (-1.30 to -0.22), p = 0.006
**Engaging in screen-based sedentary behaviour on weekdays**						
No	310	38.5%	301	38.4%	NA	NA
Yes	496	61.5%	482	61.6%	1.04 (0.82 to 1.32), p = 0.748	0.99 (0.78 to 1.27), p = 0.953
**Engaging in screen-based sedentary behaviour on weekend days**						
No	172	21.3%	125	16.0%	NA	NA
Yes	634	78.7%	658	84.0%	0.70 (0.52 to 0.93), p = 0.014	0.60 (0.44 to 0.82), p = 0.001
**Engaging in active sports, dance, or games at least once in previous weekend**						
No	144	17.9%	199	25.4%	NA	NA
Yes	662	82.1%	584	74.6%	1.58 (1.23 to 2.04), p<0.001	1.47 (1.10 to 1.96), p = 0.009
**Objectively measured sedentary time (minutes/24 hours)**	669	461.97 (98.28)	645	468.64 (93.01)	-8.45 (-30.69 to 13.80), p = 0.457	-6.26 (-27.26 to 14.73), p = 0.559
**Parent-reported sedentary time (minutes/24 hours)**	814	202.74 (125.23)	779	217.61 (132.87)	-12.63(-26.73 to 1.46), p = 0.079	-14.71 (-29.54 to 0.11), p = 0.052
**Objectively measured MVPA time (minutes/24 hours)**	669	63.98 (32.52)	645	62.65 (27.54)	3.24 (-3.46 to 9.94), p = 0.343	0.56 (-5.32 to 6.43), p = 0.853
**Parent-reported MVPA time (minutes/24 hours)**	816	132.86 [90.00–184.29]	779	126.43 [90.00–188.57]	1.20 (-9.44 to 11.85), p = 0.825	0.50 (-10.64 to 11.65), p = 0.929
**Achieving ≥60 minutes MVPA in 24 hours (objectively measured)**^**h**^						
No	353	52.8%	337	52.3%	NA	NA
Yes	316	47.2%	308	47.8%	1.16 (0.69 to 1.95), p = 0.564	1.02 (0.61 to 1.68), p = 0.954
**Achieving ≥60 minutes MVPA in 24 hours (parent reported)**^**h**^						
No	58	8.2%	58	8.5%	NA	NA
Yes	646	91.8%	622	91.5%	0.97 (0.64 to 1.46), p = 0.883	0.78 (0.49 to 1.22), p = 0.273
**Psychosocial outcomes**						
**PedsQL total score**	806	85.86 [77.17–92.39]	783	83.69 [76.08–91.30]	1.27 (-0.13 to 2.67), p = 0.076	1.16 (-0.36 to 2.69), p = 0.134
**CHU9D utility score**	806	0.94 (0.06)	781	0.93 (0.07)	0.008 (0.000 to 0.015), p = 0.034	0.007 (0.000 to 0.016), p = 0.056
**Kidscreen-52 bullying**	806	13.94 (1.64)	783	13.73(1.82)	0.136 (-0.085 to 0.359), p = 0.228	0.090 (-0.134 to 0.314), p = 0.431

^a^*N* = the total number of children from whom we collected valid data at the follow-up.

^b^Baseline adjusted = adjusted for baseline outcome and school clustering.

^c^Further adjusted = adjusted for baseline outcome, prespecified school-level (i.e., whether the school provides midmorning snack, whether the school has an indoor activity room) and child-level sociodemographic (i.e., age, sex, and mother education level) and behavioural (daily average servings of fruit and vegetables, weekly servings of unhealthy snacks and sugar-added drink, objectively measured time in MVPA [minutes/24 hours] and objectively measured sedentary time [minutes/24 hours]) covariates.

^d^Intracluster correlation coefficients of the primary outcome at the follow-up were 0.118 (0.054 to 0.240) and 0.112 (0.057 to 0.211), respectively, in the baseline-adjusted and further-adjusted analyses.

^e^Weight status was defined according WHO growth charts (BMI z score) cut off points.

^f^Based on children who provided valid data.

^g^Adjusted for baseline daily average servings of fruit and vegetables.

^h^Adjusted for baseline objectively measured time in MVPA (per 24 hours).

**Abbreviations:** BMI, body mass index; CHU9D, Child Health Utility 9D; IQR, interquartile range; MD, mean difference; MVPA, interquartile range; OR, odds ratio; PedsQL, Pediatric Quality of Life Inventory

### Secondary outcomes

For the secondary outcomes (Table 3), daily intake and the proportion of children consuming at least 5 daily portions of fruit and vegetables were significantly higher in the intervention than the control group (baseline-adjusted MD = 0.33, 95% CI: 0.14 to 0.52, *p* = 0.001). Weekly consumption of sugar-sweetened beverages and unhealthy snacks was significantly lower in the intervention than the control group (baseline-adjusted MD = −0.81, 95% CI: −1.42 to −0.20, *p* = 0.010). The proportion of children engaging in screen-based sedentary behaviour at the weekend was lower (baseline-adjusted OR = 0.70, 95% CI: 0.52 to 0.93, *p* = 0.014) whilst the proportion of children engaging in active sports, dance, or games at least once at the weekend was higher (baseline-adjusted OR = 1.58, 95% CI: 1.23 to 2.04, *p* < 0.001) in the intervention than the control group. Parent-reported average minutes of sedentary time was lower in the intervention compared with the control group at borderline statistical significance (baseline-adjusted MD = −12.63, 95% CI: −26.73 to 1.46, *p* = 0.079). We also found a favourable difference between the groups in the proportion with overweight or obesity at borderline statistical significance (baseline-adjusted OR = 0.53, 95% CI: 0.27 to 1.05, *p* = 0.067). The mean waist circumference was significantly lower in the intervention than control group in the further-adjusted model (MD = −0.53, 95% CI: −1.06 to −0.01, *p* = 0.047) but not in the baseline-adjusted model (MD = −0.37, 95% CI: −0.85 to 0.11, *p* = 0.128). For psychosocial outcomes, we found a significant favourable difference between the groups in the CHU9D utility score (baseline-adjusted MD = 0.008, 95% CI: 0.000 to 0.015, *p =* 0.034).

### Prespecified subgroup analysis

In the prespecified subgroup analysis (S2 Appendix) by child’s sex, the intervention effect on anthropometric measurements was more marked in girls compared with boys. This was seen for BMI z score (baseline-adjusted MD = −0.18, 95% CI: −0.32 to −0.05, *p* = 0.007 versus MD = −0.09, 95% CI: −0.24 to 0.05; *p* = 0.22, respectively; *p* for interaction term = 0.015), proportion overweight or obese (baseline-adjusted OR = 0.20, 95% CI: 0.06 to 0.62, *p* = 0.005 versus OR = 0.84, 95% CI: 0.41 to 1.72, *p* = 0.63, respectively; *p* for interaction term = 0.032), and waist circumference (baseline-adjusted MD = −0.69, 95% CI: −1.26 to −0.12, *p* = 0·017 versus MD = −0.10, 95% CI: −0.68 to 0.48, *p* = 0.733, respectively; *p* for interaction term = 0.001). For other outcomes, we found no evidence that the intervention worked differently in girls and boys. The intervention effect was also greater among children who were overweight or obese at baseline, compared with those who were not, on BMI z score (baseline-adjusted MD = −0.49, 95% CI: −0.73 to −0.25, *p* < 0·001 versus MD = −0.06, 95% CI: −0.18 to 0.06, *p* = 0.310, respectively; *p* for interaction term < 0.001); and waist circumference (baseline-adjusted MD = −1.38, 95% CI: −2.23 to −0.53, *p* = 0.001 versus MD = −0.12, 95% CI: −0.60 to 0.36, *p* = 0.616, respectively; *p* for interaction term < 0.001). Subgroup analyses by mother’s education level showed that the intervention effect on the proportion of children engaging in active sports, dance, or games at least once at the weekend was greater in children whose mothers had higher versus lower education (baseline-adjusted OR = 1.72, 95% CI: 1.22 to 2.43, *p* = 0.002 versus OR = 1.47, 95% CI: 1.00 to 2.15, *p* = 0.048; *p* for interaction term = 0.008).

### Process evaluation

Intervention delivery was achieved with high fidelity. We successfully delivered all the intended intervention activities and content. The duration of sessions was as planned for 95.2% of the intervention activities targeting children and 79.9% of the interventions activities targeting families. Attendance rates for school-based intervention activities were also high: 97.9% to 99.3% for those targeting children and 88.1% for those targeting family members. Child engagement with (assessed by self-monitoring cards) and parental support for health behaviour challenges (assessed by parent written feedback on child performance), and attendance of family members for all workshops were higher among girls (compared with boys), children with overweight/obesity (compared with those who were not), and children whose mothers had higher education (compared with lower education). Furthermore, interviews with parents and teachers suggested that girls were more engaged during the intervention activities and were more likely to complete the challenges that were set within the activities. Illustrative quotes are presented in Box 1.

**Box 1. Quotes from study participants**

- ‘I had discussed this with other parents…girls were better (in health behaviour challenges)…they take the challenges more seriously. At home, they show a strong determination in completing tasks. Boys, on the other hand, are more casual; they usually do it when they feel like it. Additionally, they may not adhere to tasks (challenges).’—Parent ID number 12
- ‘Boys are less attentive (in intervention workshops), while girls are usually well disciplined and more attentive. As a result, girls usually understand and achieve better in health challenges.’—Teacher ID number 26

### Economic analysis

**Fig 2:**
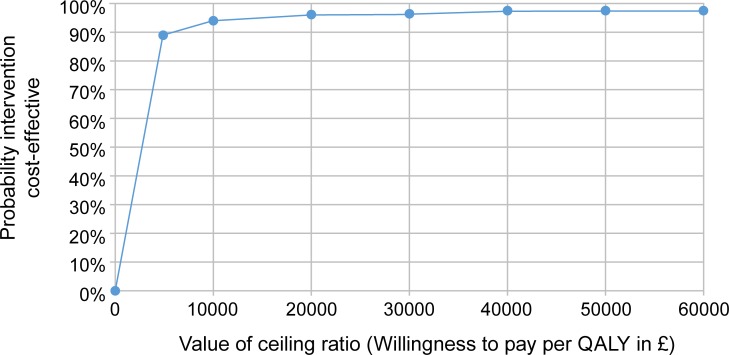
CEAC. CEAC, Cost Effectiveness Acceptability Curve; QALY, quality-adjusted life year.

Complete cost and outcome data were available for >95% of children, thus no imputation was needed. Assuming an average class size of 45, the incremental cost of the intervention was 35.53 Yuan (£7.04/ US$10.01) per child. QALY MD between groups was 0.004 (95% CI: 0.000–0.007; *p* = 0.034) and 0.004 (95% CI: 0.000 to 0.008; *p* = 0.056), in the baseline and further-adjusted models, respectively. The ICER was £1,760 (US$2,502) per QALY, which is far below the £20,000 per QALY and $50,000 per QALY thresholds for cost effectiveness in the UK and US, respectively. The ICER was £54 (US$77)/BMI z score change. The CEAC (Fig 2) shows a 95% probability of the intervention being cost effective at a willingness to pay threshold of £20,000 per QALY. Even at a willingness to pay threshold of £5,000 per QALY, the probability of cost effectiveness remains high at 90%.

## Discussion

In this cluster-randomised controlled trial of a theoretically developed, school- and family-based obesity prevention programme targeting 6- to 7-year-old Chinese children, delivered over 12 months, we found a significant favourable intervention effect on reducing BMI z scores, which was also highly cost effective. Previous research in children has shown clinical benefits with BMI z score differences as small as 0.1 units [25], and in the context of prevention, the magnitude of the effect observed for this intervention is likely to be clinically important. We also found evidence of beneficial effects on children’s waist circumference and dietary, physical activity, and sedentary behaviours. There was evidence that the intervention was particularly effective in girls and children who were overweight/obese at baseline. These subgroup differences were supported by the findings from the process evaluation in terms of differential engagement with intervention activities.

This is one of the largest trials of childhood obesity prevention to date. To our knowledge, this study provides the first example of a rigorously developed and evaluated childhood obesity prevention trial, not only in Asia but in LMIC settings. This trial also addressed a number of methodological limitations highlighted by the latest Cochrane review [26] of school-based obesity prevention trials conducted predominantly in high-income countries and included an economic evaluation. We developed the 12-month, multicomponent intervention using the framework set out by the UK MRC [8]. The intervention comprised elements identified as promising in international and Chinese systematic reviews and incorporated a range of behaviour-change techniques and social marketing principles [6]. We published the trial protocol and statistical analysis plan prospectively. We sought to reduce risk of bias by using objective measurements where possible, ensuring baseline measurements were undertaken prior to randomisation, and using assessors who were masked to allocation for follow-up measurements. Loss to follow-up was very low, with 96.7% of the randomised children and all randomised schools retained at the primary follow-up. Analyses took account of school clustering. We achieved high intervention delivery adherence and very good engagement from children and their family members. Our process evaluation allowed us to contextualise and interpret the main trial findings more appropriately.

Nevertheless, there were also limitations. Although this is one of the first major childhood obesity prevention trials in China to include objective measures of physical activity and body fat percent, usable data from those measurements was low (just over 50%). We did not account for missing data, because this is unlikely to change the overall results of this study due to the small amount of missing data on the primary and all other secondary outcomes. For the primary outcome (BMI z score), missing data were less than 5%. For all other secondary outcomes, we successfully collected data on 85.9% to 96.8% of children, which was higher than the level achieved by previous trials of a similar size. Moreover, supplementary subjective measurements of physical activity and other objective measures of adiposity showed similar overall effects, suggesting the findings are likely to be valid. Because of the lack of validated dietary assessment instruments for Chinese children, we adapted tools developed and validated for English children [12]. We had previously used similar adapted tools to assess dietary behaviours of Chinese children living in the same city [23]. The primary and the majority of the secondary outcomes were measured by objective and/or validated instruments. However, the measures for some of the physical activity–related secondary outcomes (e.g., child’s engagement in active games in the weekend) were constructed by the research team for use in the trial. Whilst the validity of these self-designed measures is unknown, results from these measures were in line with the findings from other physical activity measures obtained by objective and/or validated instruments. Furthermore, because of the nature of the intervention, it was impossible to mask participating schools and individuals to group allocation. Also, for the economic analysis, a judgement of cost effectiveness had to be made using established thresholds from a high-income country setting, and it is unclear if China has similar societal values.

Findings of this trial added to the current knowledge related to the effectiveness of childhood obesity prevention interventions within a global context in 2 ways. First, the latest Cochrane review (published in 2019) found that for trials aimed at children aged 6 to 12 years, evaluating multicomponent interventions targeting both dietary and physical activity behaviours, the standardised MD in adiposity (measured as BMI or BMI z score) between the intervention and control groups was −0.05 (95% CI −0.10 to −0.01) [26]. This summary estimate was based on low certainty evidence, and it is important to know that a much bigger effect size could be achieved in a trial that was well designed and implemented. Second, this trial was conducted in an Asian, middle-income country, whereas 89% of the trials included in the Cochrane review were conducted in high-income countries. Only 1 Asian trial, judged by the reviewers as poorly reported, was included, reporting an ineffective physical activity intervention in preschool children in Thailand (*n* = 292 from 2 nurseries). A more recent systematic review of childhood obesity prevention trials in Asia [5] again highlighted the lack of high-quality trials in this part of the world.

This trial demonstrated favourable effects on a range of anthropometric measurements as well as on health behaviours. A systematic review of childhood obesity prevention interventions in high-income countries [27] identified at least moderate strength of evidence for the effectiveness of school-based interventions. Interventions implemented in schools with a home element were most likely to achieve favourable results. Our findings showed that school-based interventions with a family component in a middle-income country is also likely to achieve a favourable intervention effect.

However, our findings differ from those reported in 3 large, well-conducted childhood obesity prevention trials in the UK, which have been published in the last 3 years and since the publication of the Cochrane review. These trials found no evidence for the effectiveness of school-based prevention interventions [28–30], even though one of these trials [28] was conducted by our research team using similar development and evaluation methodology. This highlights the importance of ‘context’ in determining intervention effectiveness. We propose the following potential explanations for such differences. First, such prevention interventions may be more effective in countries where the obesity epidemic is at an earlier stage and there are fewer existing initiatives implemented by the government, local authorities, or schools. Moreover, the western diet and modern life style, though growing in importance, have not yet become the norm in China, which might have made acceptance of healthier alternatives easier. Second, although those UK-based interventions were also well developed, the CHIRPY DRAGON trial achieved a higher level of intervention adherence. We successfully delivered all intervention activities as planned, with a very high proportion of consented children and targeted family members participating in the intervention activities. In this trial, we used dedicated staff employed specifically to deliver all intervention activities, whilst most previous school-based interventions were delivered by school teachers. It has been previously noted that obesity prevention programmes are more effective when delivered by dedicated staff versus classroom teachers [31]. Whilst this may be less feasible in high-income settings, the staff employed to deliver the interventions in this trial were well accepted by schools. Furthermore, we received support from local authorities with recruitment and during intervention delivery. Involvement of local authorities was also identified as key to successful intervention implementation in another Chinese trial [32]. Finally, a more hierarchical structure and respect for schools and teachers in LMIC compared with high-income countries may have further supported intervention implementation.

In prespecified subgroup analyses, we found some evidence that the CHIRPY DRAGON programme was particularly effective in girls. This is in keeping with findings from previous Chinese trials [33,34] and meta-analyses of childhood obesity interventions in high-income countries that examined gender as a moderator of intervention effect [35]. This has important implications for China, where the rate of increase in obesity has been particularly marked in boys compared with girls [2] and suggests that identification of effective interventions targeting boys is a priority.

We believe our findings provide novel and important information for researchers, policy makers, and research funders in other countries. First, several countries share the same language (e.g., Mandarin is commonly used as a first language in Malaysia and Singapore), foods/dietary habits, and cultures (e.g., a preference for boys and living in 3-generation households in which grandparents have an important role in feeding the child) with China. In fact, the problem of grandparent care in relation to childhood obesity is also common in non-Asian countries [36]. The intervention we tested in this study is the first childhood obesity prevention programme that included a substantial component aimed at improving grandparents’ childcare knowledge and skills. In this respect, we believe that our results provide useful and new information for countries that share the same problem. Second, as mentioned above, our research team had conducted 2 trials with a similar design using the same robust framework recommended by the MRC. However, evidence of effectiveness was found in the one conducted in a middle-income country setting (China) but not in the one conducted in a high-income country (UK). This difference is likely to be of some interest to researchers, policy makers, and research funders.

This study has shown that an evidence-based obesity prevention programme delivered through schools with high implementation adherence may be effective in reducing the emerging epidemic of childhood obesity in China and offer a cost-effective use of public resources. Future research should identify strategies to enhance beneficial effects among boys and investigate the transferability of the intervention to other provinces in China and countries that share the same language and cultures.

## Supporting information

S1 Appendix A timeline cluster diagram to assess risk of bias. (DOCX)

S2 Appendix Results of prespecified subgroup analysis. (DOCX)

S1 Table Summary of the CHIRPY DRAGON intervention components and delivery. CHIRPY DRAGON, Chinese Primary School Children Physical Activity and Dietary Behaviour Changes Intervention
(DOCX)

S2 Table Summary of data collection methods. (DOCX)

S3 Table Comparison of trial completers (*n* = 1,586) and those lost to follow up (*n* = 55) by baseline characteristics. (DOCX)

S4 Table CONSORT checklist of information to include when reporting a cluster-randomised trial. (DOCX)

## Abbreviations

BMI: body mass index
CEA: cost-effectiveness analysis
CEAC: Cost Effectiveness Acceptability Curve
CHIRPY DRAGON: Chinese Primary School Children Physical Activity and Dietary Behaviour Changes Intervention
CHU 9D: Child Health Utility 9D
CUA: cost-utility analysis
GDP PPP: Gross Domestic Product Purchasing Power Parity
ICER: incremental cost-effectiveness ratio
IQR: interquartile range
LMIC: low- and middle-income countries
MD: mean difference
MRC: Medical Research Council
MVPA: moderate to vigorous physical activity
OR: odds ratio
PedsQL: Pediatric Quality of Life Inventory
QALY: quality-adjusted life year
