# Supplementary material for: The CHIRPY DRAGON intervention in preventing obesity in Chinese primary-school--aged children: A cluster-randomised controlled trial
Source: PLoS Med. 2019 Nov 26;16(11):e1002971. doi: 10.1371/journal.pmed.1002971 (PMC6879117; doi:10.1371/journal.pmed.1002971)
Supplement: S1 Appendix — (DOCX) [file pmed.1002971.s001.docx]

**S1 Appendix: A timeline cluster diagram to assess risk of bias**

TRIAL PROCESS

Outcome assessment 1

8

Usual care

7b

Intervention delivery

7a

**Randomisation**

**6**

Baseline assessment

5

Recruitment

4

Identification

3

Recruitment

2

Identification

1

The rings and stick figures refer to the stages that involve schools (clusters) and children (individuals), respectively. A stage involving school level only, is represented by a ring only, while a stage involving children only is represented by a stick figure only. Stages involving children and schools are presented by both rings and stick figures. The shading of the boxes background indicates the level of blinding present. Boxes with dark background indicates stages with complete blinding while grey refers to partial blinding, and white to no blinding. Points that are separated by distinct boxes for interaction and control arms present stages that differ between the trial arms.

| **1** | **Cluster identification**  All non-boarding, state-funded primary schools located in the traditional urban districts of Guangzhou city (n=353) were eligible for inclusion. A trial statistician randomly selected 40 schools, using a random number generator. They were the schools to be invited to take part in the study from the first week of September in 2015 (randomization occurred in January 2016, post baseline assessment, as detailed below). |
| --- | --- |
| **2** | **Cluster recruitment**  Through support from local education and health authorities (an official support letter was sent to each of the sampled schools) and personal visits (with written information sheet/consent form) or telephone communication from the research team members, all 40 schools agreed to take part in the trial within 1-2 weeks. |
| **3** | **Participant identification**  All children from Year-One classes (6-7 years) within the consented schools were eligible. A research team member randomly selected one Year-One class from each school to take part in outcome measures, during a personal visit to the school. |
| **4** | **Participant recruitment**  The research team members distributed invitation letters, information sheets and consent forms to the head teacher of each selected class. A copy of those documents were included in an envelope for each eligible child, labelled ‘a letter to the family’. The head teachers passed on those envelopes to individual children who then delivered the envelopes to their parents/guardians. Parents/guardians of the children were advised: 1) to inform the research team if they believed there were any medical reasons that a child should not participate in any outcome measures and/or intervention activities, and that 2) they could withdraw from this study at any time. Children who returned a completed consent form signed by their parents/guardians took part in outcome measures. |
| **5** | **Participant and cluster baseline assessment**  Baseline measurements were taken (September – December 2015) when participating children were in Year One (6-7 years). Children’s date of birth and gender were obtained from parents’ questionnaires and validated with school admission records. Assessments were undertaken by independent and trained assessors who were blinded to school allocation using standardised procedures and validated or adapted instruments. Parents and other live-in adult family members of the children were asked to complete questionnaires covering the dietary, sedentary, physical and sleep activity habits of the child and the family, quality of life and household expenditure. Children were invited to 1) complete a 24-hour diary (on health-related behaviour) and a questionnaire (on family life style and psychosocial measures), and 2) wear a wrist-worn accelerometer continuously for five days (including three weekdays and a weekend). These child based measures were also administered through independent trained research staff who were blinded to school allocation.  School level data was collected from questionnaires administered to head teachers or a nominated representative. |
| **6** | **Randomisation**  Randomisation took place in January 2016, after baseline assessment. An UK-based medical statistician performed stratified randomisation using the ralloc command in Stata to allocate the 40 schools (clusters) to intervention (n=20) and control (n=20) arms. Four strata were generated based on whether the school provided mid-morning snacks or had an indoor activity room. |
| **7a** | **Intervention delivery**  The intervention took place between March 2016 and March 2017. No blinding for children, family members or teachers. Process evaluation was carried out throughout the intervention year using a range of methods, including implementation record forms, child self-monitoring fun cards, direct observation by non-delivery staff, focus groups and interviews. |
| **7b** | **Usual care**  No blinding for children, family members or teachers. |
| **8** | **Outcome assessment – primary follow up**  Follow-up measurements were undertaken (April-July 2017) immediately upon the completion of the intervention period, when participating children were in Year Two (7-8 years). Assessments were undertaken by independent trained research staff who were blinded to school allocation using standardised procedures and validated or adapted instruments. Parents and other live-in adult family members of the children were asked to complete questionnaires covering the dietary, sedentary, physical and sleep activity habits of the child and the family, quality of life and household expenditure. Children were invited to 1) complete a 24-hour diary (on health-related behaviour) and a questionnaire (on family life style and psychosocial measures), and 2) wear a wrist-worn accelerometer continuously for five days (including three weekdays and a weekend). These child based measures were also administered through independent trained research staff who were blinded to school allocation. |
