# Supplementary material for: The CHIRPY DRAGON intervention in preventing obesity in Chinese primary-school--aged children: A cluster-randomised controlled trial
Source: PLoS Med. 2019 Nov 26;16(11):e1002971. doi: 10.1371/journal.pmed.1002971 (PMC6879117; doi:10.1371/journal.pmed.1002971)
Supplement: S2 Appendix — (DOCX) [file pmed.1002971.s002.docx]

**S2 Appendix: Results of Pre-specified Sub group Analysis**

**List of Tables A to I in S2 Appendix.**

- Table A: Adjusted differences for the primary and secondary outcomes (anthropometric, diet, physical activity and psychological variables) between control and intervention groups in girls at follow up
- Table B: Adjusted differences for the primary and secondary outcomes (anthropometric, diet, physical activity and psychological variables) between control and intervention groups in boys at follow up
- Table C: Adjusted differences for the primary and secondary outcomes (anthropometric, diet, physical activity and psychological variables) between control and intervention groups at follow up - P-values for interaction terms for sex
- Table D: Adjusted differences for the primary and secondary outcomes (anthropometric, diet, physical activity and psychological variables) between control and intervention groups at follow up (in children who were non overweight at baseline)
- Table E: Adjusted differences for the primary and secondary outcomes (anthropometric, diet, physical activity and psychological variables) between control and intervention groups at follow up (in children who were overweight or obese at baseline)
- Table F: Adjusted differences for the primary and secondary outcomes (anthropometric, diet, physical activity and psychological variables) between control and intervention groups at follow up - P-values for interaction terms for child baseline weight status
- Table G: Adjusted differences for the primary and secondary outcomes (anthropometric, diet, physical activity and psychological variables) between control and intervention groups at follow up (in children whose mothers did not have higher education)
- Table H: Adjusted differences for the primary and secondary outcomes (anthropometric, diet, physical activity and psychological variables) between control and intervention groups at follow up (in children whose mothers had higher education)
- Table I: Adjusted differences for the primary and secondary outcomes (anthropometric, diet, physical activity and psychological variables) between control and intervention groups at follow up - P-values for interaction terms for mother education level

**Table A: Adjusted differences for the primary and** **secondary outcomes (anthropometric, diet, physical activity and psychological variables) between control and intervention groups in girls at follow up**

|  | **Intervention group** | | **Control group** | | **Mean difference or Odds ratio**  **(95% CI), P value** | |
| --- | --- | --- | --- | --- | --- | --- |
|  | **N** ^a^ | **Mean (SD)/** **Median [IQR] ^b^/ %** | **N** ^a^ | **Mean (SD)/** **Median [IQR] ^b^/%** | **Baseline adjusted^c^**  **(primary analysis)** | **Further adjusted ^d^**  **(secondary analysis)** |
| **Adiposity related outcomes** | | | | | | |
| BMI z score | 362 | -0.60 (1.02) | 363 | -0.41 (1.21) | -0.18 (-0.32 to -0.05), p=0.007 | -0.17 (-0.29 to -0.05), p=0.005 |
| Obese/Overweight **^e^** |  |  |  |  |  |  |
| No | 331 | 91.4% | 311 | 85.7% | NA | NA |
| Yes | 31 | 8.6% | 52 | 14.3% | 0.2 (0.1 to 0.6), p=0.005 | 0.3 (0.1 to 0.9), p=0.043 |
| Waist circumference | 363 | 56.04 (6.02) | 366 | 56.98 (6.48) | -0.69 (-1.26 to -0.12), p=0.017 | -0.65 (-1.26 to 0.04), p=0.038 |
| Body fat % **^f^** | 214 | 20.25 (5·48) | 197 | 21.44 (5·01) | -0.01 (-0·03 to 0·01), p=0·17 | -0.01 (-0.03 to 0.00), p=0.14 |
| **Blood pressure** | | | | | | |
| Systolic blood pressure (mmHg) | 363 | 100.19 (8.26) | 366 | 100.21 (8.96) | -0.08(-1.43 to 1.27), p=0.91 | 0.08 (-1.24 to 1.41) , p=0.90 |
| Diastolic blood pressure (mmHg) | 363 | 60.73 (6.96) | 366 | 61.08 (7.34) | -0.59 (-2.02 to 0.84), p=0.42 | -0.49 (-2.01 to 1.02), p=0.52 |
| **Behavioral outcomes** | | | | | | |
| Daily average servings of fruit and vegetables | 294 | 3.0 [2.0 -4.0] | 300 | 3.0 [2.0-4.0] | 0.3 (0.1 to 0.6), p=0.003 | 0.4 (0.2 to 0.7), p=0.001 |
| ≥ 5 portions of fruit and vegetables **^g^** |  |  |  |  |  |  |
| No | 289 | 80.7% | 330 | 91.7% | NA | NA |
| Yes | 69 | 19.3% | 30 | 8.3% | 2.4 (1.5 to 3.9), p<0.001 | 3.1 (1.8 to 5.5), p<0.001 |
| Weekly average servings of unhealthy snacks and sugar added drinks | 294 | 1.0 [0.0-2.5] | 300 | 2.5 [1.0-4.5] | -1.2 (-1.9 to -0.4), p=0.003 | -1.00 (-1.8 to -0.2), p=0.018 |
| Engaging in screen-based sedentary behavior on weekdays |  |  |  |  |  |  |
| No | 150 | 41.7% | 166 | 45.2% | NA | NA |
| Yes | 210 | 58.3% | 201 | 54.8% | 1.2 (0.8 to 1.6), p=0.35 | 1.1 (0.8 to 1.6), p=0.51 |
| Engaging in screen-based sedentary behavior on weekend days |  |  |  |  |  |  |
| No | 89 | 24.7% | 69 | 18.8% | NA | NA |
| Yes | 271 | 75.3% | 298 | 81.2% | 0.7 (0.5 to 1.1), p=0.11 | 0.7 (0.5 to 1.1), p=0.14 |
| Engaging in active sports, dance, or games at least once in previous weekend |  |  |  |  |  |  |
| No | 61 | 16.9% | 84 | 22.9% | NA | NA |
| Yes | 299 | 83.1% | 283 | 77.1% | 1.5 (1.0 to 2.1), p=0.05 | 1.4 (0.9 to 2.2), p=0.11 |
| Objectively measured sedentary time (minutes/24 hours) | 311 | 463.3 (99.1) | 304 | 478.3 (93.7) | -19.8 (-45.7 to 6.1), p=0.13 | -9.5 (-33.7 to 14.74), p=0.44 |
| Parent reported sedentary time (minutes/24 hours) | 353 | 204.5 (120.8) | 359 | 219.3 (138.9) | -13.3 (-32.4 to 5.7), p=0.17 | -12.4 (-34.14 to 9.41), p=0.27 |
| Objectively measured MVPA time (minutes/24 hours) | 311 | 55.3 (27.9) | 304 | 54.0 (24.9) | 6.0 (-1.3 to 13.1), p=0.11 | 2.4 (-4.0 to 8.8), p=0.46 |
| Parent reported MVPA time (minutes/24 hours) | 294 | 124.3 [84.3-175.7] | 300 | 115.7 [85.7-174.6] | 0.8 (-15.5 to 17.1), p=0.92 | 4.5 (-13.9 to 22.9), p=0.63 |
| Achieving ≥ 60 minutes MVPA in 24 hours (objectively measured) **^h^** |  |  |  |  |  |  |
| No | 196 | 63.0% | 204 | 67.1% | NA | NA |
| Yes | 115 | 37.0% | 100 | 32.9% | 2.1 (1.0 to 4.3), p=0.043 | 1.7 (0.7 to 4.1), p=0.21 |
| Achieving ≥ 60 minutes MVPA in 24 hours (parent reported) **^h^** |  |  |  |  |  |  |
| No | 31 | 10.0% | 31 | 9.9% | NA | NA |
| Yes | 279 | 90.0% | 281 | 90.1% | 0.9 (0.5 to 1.5), p=0.65 | 0.8 (0.4 to 1.5), p=0.43 |
| **Psycho-social outcomes** | | | | | | |
| PedsQL total score | 360 | 86.9 [78.2-93.4] | 367 | 84.7 [77.1- 92.3] | 1.4 (-0.7 to 3.5), p=0.19 | 1.00 (-1.2 to 3.2), p=0.36 |
| CHU9D utility score | 360 | 0.942 (0.059) | 367 | 0.934 (0.063) | 0.007 (-0.002 to 0.017),p=0.15 | 0.007 (-0.003 to 0.018), p=0.18 |
| Kidscreen-52 bullying | 360 | 13.95 (1.67) | 367 | 13.77 (1.69) | 0.09 (-0.18 to 0.37), p=0.51 | 0.09 (-0.20 to 0.38), p=0.54 |

a: N=the total number of children from whom we collected valid data at the follow up. b: IQR= interquartile range. c: baseline adjusted= adjusted for baseline outcome and school clustering. d: further adjusted=adjusted for baseline outcome, pre-specified school- level (i.e. whether the school provides mid-morning snack, whether the school has an indoor activity room) and child-level socio-demographic (i.e. age, sex and mother education level) and behavioural [daily average servings of fruit and vegetables, weekly servings of unhealthy snacks and sugar added drink, objectively measured time in MVPA (minutes/24 hours) and objectively measured sedentary time (minutes/24 hours)] covariates. e: weight status was defined according WHO growth charts (BMI z score) cut off points. f: based on children who provided valid data. g: adjusted for baseline daily average servings of fruit and vegetables. h: adjusted for baseline objectively measured time in MVPA (per 24 hours).

**Table B: Adjusted differences for the primary and secondary outcomes (anthropometric, diet, physical activity and psychological variables) between control and intervention groups in boys at follow up**

|  | **Intervention group** | | **Control group** | | **Mean difference or Odds ratio**  **(95% CI), P value** | |
| --- | --- | --- | --- | --- | --- | --- |
|  | **N** ^a^ | **Mean (SD)/** **Median [IQR] ^b^/%** | **N** ^a^ | **Mean (SD)/** **Median [IQR]^b^ /%** | **Baseline adjusted^c^**  **(primary analysis)** | **Further adjusted ^d^**  **(secondary analysis)** |
| **Adiposity related outcomes** | | | | | | |
| BMI z score | 442 | -0.15 (1.33) | 414 | -0.07 (1.44) | -0.09 (-0.24 to 0.05), p=0.22 | -0.11 (-0.27 to 0.05), p=0.18 |
| Obese/Overweight **^e^** |  |  |  |  |  |  |
| No | 348 | 78.7% | 320 | 77.3% | NA | NA |
| Yes | 94 | 21.3% | 94 | 22.7% | 0.8 (0.4 to 1.7), p=0.63 | 0.9 (0.4 to 2.1), p=0.85 |
| Waist circumference | 422 | 58.61 (7.22) | 415 | 58.61 (7.11) | -0.10 (-0.68 to 0.48), p=0.73 | -0.39 (-1.08 to 0.29), p=0.26 |
| Body fat % **^f^** | 262 | 17.91 (5·56) | 234 | 18.69 (5·84) | -0.01 (-0·03 to 0·01), p=0·19 | -0.01 (-0.03 to 0.01), p=0.22 |
| **Blood pressure** | | | | | | |
| Systolic blood pressure (mmHg) | 442 | 102.65 (8.86) | 415 | 102.32 (8.91) | -0.03(-1.68 to 1.61), p=0.97 | -0.54 (-2.25 to 1.16) , p=0.53 |
| Diastolic blood pressure (mmHg) | 442 | 60.89 (7.31) | 415 | 61.34 (7.03) | -0.64 (-2.21 to 0.93), p=0.42 | -0.78 (-2.3 to 0.74), p=0.32 |
| **Behavioral outcomes** | | | | | | |
| Daily average servings of fruit and vegetables | 376 | 3.0 [2.0-4.0] | 344 | 3.0 [2.0-3.9] | 0.3 (0.1 to 0.6), p=0.018 | 0.3 (0.0 to 0.5), p=0.032 |
| ≥ 5 portions of fruit and vegetables **^g^** |  |  |  |  |  |  |
| No | 364 | 83.3% | 366 | 89.5% | NA | NA |
| Yes | 73 | 16.7% | 43 | 10.5% | 1.7 (1.1 to 2.7), p=0.021 | 1.5 (0.9 to 2.5), p=0.11 |
| Weekly average servings of unhealthy snacks and sugar added drinks | 376 | 1.0 [0.0 -3.5] | 344 | 2.0 [0.0-3.5] | -0.6 (-1.5 to 0.4), p=0.25 | -0.5 (-1.2 to 0.2), p=0.16 |
| Engaging in screen-based sedentary behavior on weekdays |  |  |  |  |  |  |
| No | 160 | 35.9% | 135 | 32.5% | NA | NA |
| Yes | 286 | 64.1% | 281 | 67.5% | 0.9 (0.7 to 1.2), p=0.52 | 0.9 (0.6 to 1.2), p=0.46 |
| Engaging in screen-based sedentary behavior on weekend days |  |  |  |  |  |  |
| No | 83 | 18.6% | 56 | 13.5% | NA | NA |
| Yes | 363 | 81.4% | 360 | 86.5% | 0.7 (0.5 to 1.0), p=0.027 | 0.5 (0.3 to 0.8), p=0.001 |
| Engaging in active sports, dance, or games at least once in previous weekend |  |  |  |  |  |  |
| No | 83 | 18.6% | 115 | 27.6% | NA | NA |
| Yes | 363 | 81.4% | 301 | 72.4% | 1.7 (1.2 to 2.4), p=0.001 | 1.5 (1.0 to 2.2), p=0.042 |
| Objectively measured sedentary time (minutes/24 hours) | 358 | 460.8 (97.7) | 341 | 460.0 (91.7) | 1.0 (-23.5 to 25.4), p=0.94 | -1.8 (-25.2 to 21.6), p=0.88 |
| Parent reported sedentary time (minutes/24 hours) | 427 | 201.3 (128.9) | 402 | 216.1 (127.4) | -12.2 (-31.3 to 6.9), p=0.21 | -17.6 (-37.3 to 2.2), p=0.081 |
| Objectively measured MVPA time (minutes/24 hours) | 358 | 71.5 (34.4) | 341 | 70.4 (27.6) | 1.6 (-6.0 to 9.2), p=0.68 | 0.2 (-6.8 to 7.2), p=0.96 |
| Parent reported MVPA time (minutes/24 hours) | 376 | 137.1 [96.1-188.6] | 344 | 137.1 [90.0-197.1] | 0.6 (-11.3 to 12.5), p=0.92 | -3.2 (-16.7 to 10.3), p=0.64 |
| Achieving ≥ 60 minutes MVPA in 24 hours (objectively measured) **^h^** |  |  |  |  |  |  |
| No | 157 | 43.9% | 133 | 39.0% | NA | NA |
| Yes | 201 | 56.2% | 208 | 61.0% | 0.8 (0.5 to1.4), p=0.43 | 0.7 (0.4 to 1.3), p=0.26 |
| Achieving ≥ 60 minutes MVPA in 24 hours (parent reported) **^h^** |  |  |  |  |  |  |
| No | 83 | 18.6% | 27 | 7.3% | NA | NA |
| Yes | 363 | 81.4% | 341 | 92.7% | 1.0 (0.6 to 1.8), p=0.88 | 0.8 (0.4 to 1.5), p=0.48 |
| **Psycho-social outcomes** | | | | | | |
| PedsQL total score | 446 | 84.7 [76.1-91.3] | 416 | 82.6 [75.1- 90.7] | 1.2 (-0.1 to 2.6), p=0.082 | 1.2 (-0.4 to 2.9), p=0.14 |
| CHU9D utility score | 446 | 0.933 (0.058) | 414 | 0.922 (0.065) | 0.009 (0.000 to 0.018), p=0.039 | 0.008 (-0.001 to 0.017), p=0.10 |
| Kidscreen-52 bullying | 446 | 13.94 (1.64) | 416 | 13.73 (1.82) | 0.17 (-0.07 to 0.43), p=0.17 | 0.09 (-0.17 to 0.37), p=0.48 |

a: N=the total number of children from whom we collected valid data at the follow up. b: IQR= interquartile range. c: baseline adjusted= adjusted for baseline outcome and school clustering. d: further adjusted=adjusted for baseline outcome, pre-specified school- level (i.e. whether the school provides mid-morning snack, whether the school has an indoor activity room) and child-level socio-demographic (i.e. age, sex and mother education level) and behavioural [daily average servings of fruit and vegetables, weekly servings of unhealthy snacks and sugar added drink, objectively measured time in MVPA (minutes/24 hours) and objectively measured sedentary time (minutes/24 hours)] covariates. e: weight status was defined according WHO growth charts (BMI z score) cut off points. f: based on children who provided valid data. g: adjusted for baseline daily average servings of fruit and vegetables. h: adjusted for baseline objectively measured time in MVPA (per 24 hours).

**Table C: Adjusted differences for the primary and secondary outcomes (anthropometric, diet, physical activity and psychological variables) between control and intervention groups at follow up - P-values for interaction terms for sex**

| **Follow up outcome variable** | **Intervention vs control (BL adjusted)^a^** | **Intervention vs control (further adjusted)^b^** | |
| --- | --- | --- | --- |
|  | p value | p value | |
|  |  |  |  |
| BMI z score | 0.015 | 0.036 |  |
| Obese/Overweight^c^ | 0.032 | 0.040 |  |
| Waist | 0.001 | 0.015 |  |
| Body fat %^d^ | 0.005 | 0.041 |  |
| Systolic blood pressure (mmHg) | 0.010 | 0.020 |  |
| Diastolic blood pressure (mmHg) | 0.70 | 0.41 |  |
| Daily average servings of fruit and vegetables | 0.89 | 0.25 |  |
| ≥ 5 portions of fruit and vegetables^e^ | 0.42 | 0.099 |  |
| Weekly average servings of unhealthy snacks and sugar added drinks | 0.14 | 0.41 |  |
| Objectively measured sedentary time (hours/24 hours) | 0.05 | 0.05 |  |
| Parent reported sedentary time (hours/24 hours) | 0.88 | 0.71 |  |
| Engaging in screen-based sedentary behavior in the weekday | 0.17 | 0.31 |  |
| Engaging in screen-based sedentary behavior in the weekend | 0.13 | 0.54 |  |
| Engaging in active sports, dance, or games for at least once in the last weekend | 0.48 | 0.14 |  |
| Objectively measured MVPA time (minutes/24 hours) | <0.001 | <0.001 | |
| Parent reported MVPA time (minutes/24 hours) | 0.32 | 0.58 |  |
| Achieving >= 60 minutes MVPA in 24 hours(objectively measured)^f^ | 0.23 | 0.010 |  |
| Achieving >= 60 minutes MVPA in 24 hours(parent reported)^f^ | 0.17 | 0.47 |  |
| PedsQL total score | 0.021 | 0.015 |  |
| CHU9D utility score | 0.079 | 0.033 | |
| Kidscreen-52 bullying | 0.58 | 0.51 | |

BL: baseline. MVPA: moderate - vigorous physical activity. a: adjusted for baseline outcome. b: adjusted for baseline outcome, pre-specified school- level (i.e. whether the school provides mid-morning snack, whether the school has an indoor activity room) and child-level socio-demographic (i.e. age, sex and mother education level) and behavioral [daily average servings of fruit and vegetables, weekly servings of unhealthy snacks and sugar added drink, objectively measured time in MVPA (minutes/24 hours) and objectively measured sedentary time (minutes/24 hours)] covariates. c: adjusted for baseline BMI z-score. d: based on children who were measured by brand new electrodes at 1st follow up. e: adjusted for baseline daily average servings of fruit and vegetables. f: adjusted for baseline objectively measured time in MVPA (per 24 hours).

**Table D: Adjusted differences for the primary and** **secondary outcomes (anthropometric, diet, physical activity and psychological variables) between control and intervention groups at follow up (in children who were non overweight at baseline)**

|  | **Intervention group** | | **Control group** | | **Mean difference or Odds ratio**  **(95% CI), P value** | |
| --- | --- | --- | --- | --- | --- | --- |
|  | **N** ^a^ | **Mean (SD)/** **Median [IQR] ^b^ / %** | **N** ^a^ | **Mean (SD)/** **Median [IQR] ^b^/ %** | **Baseline adjusted^c^**  **(primary analysis)** | **Further adjusted ^d^**  **(secondary analysis)** |
| **Adiposity related outcomes** | | | | | | |
| BMI z score | 650 | -0.72 (0.93) | 630 | -0.67 (0.98) | -0.06 (-0.18 to 0.06), p=0.31 | -0.05 (-0.17 to 0.06), p=0.37 |
| Obese/Overweight **^e^** |  |  |  |  |  |  |
| No | 627 | 96.5% | 600 | 95.2% | NA | NA |
| Yes | 23 | 3.5% | 30 | 4.8% | 0.8 (0.4 to 1.6), p=0.50 | 1.3 (0.5 to 3.0), p=0.63 |
| Waist circumference | 651 | 55.23 (4.27) | 630 | 55.45 (4.08) | -0.12 (-0.60 to 0.36), p=0.62 | -0.25 (-0.78 to 0.26), p=0.33 |
| Body fat % **^f^** | 393 | 18.10 (5·08) | 349 | 18.88 (5.13) | -0.01 (-0·03 to 0·01), p=0.18 | -0.01 (-0.03 to 0.00), p=0.12 |
| **Blood pressure** | | | | | | |
| Systolic blood pressure (mmHg) | 651 | 100.29 (8.08) | 630 | 99.99 (8.45) | 0.15 (-1.20 to 1.50), p=0.83 | -0.01 (-1.33 to 1.31) , p=0.99 |
| Diastolic blood pressure (mmHg) | 651 | 59.97 (6.70) | 630 | 60.29 (6.69) | -0.56 (-1.81 to 0.69), p=0.38 | -0.68 (-1.93 to 0.57), p=0.29 |
| **Behavioral outcomes** | | | | | | |
| Daily average servings of fruit and vegetables | 545 | 3.0 [2.0-4.0] | 521 | 3.0 [2.0-4.0] | 0.4 (0.2 to 0.6), p<0.001 | 0.4 (0.2 to 0.6), p<0.001 |
| ≥ 5 portions of fruit and vegetables **^g^** |  |  |  |  |  |  |
| No | 526 | 82.2% | 556 | 89.8% | NA | NA |
| Yes | 114 | 17.8% | 63 | 10.2% | 1.9 (1.3 to 2.7), p=0.001 | 2.0 (1.4 to 3.0), p=0.001 |
| Weekly average servings of unhealthy snacks and sugar added drinks | 545 | 1.0 [0.0-2.5] | 521 | 2.5 [0.0-4.5] | -1.0 (-1.7 to -0.3), p=0.004 | -1.08 (-1.68 to -0.48), p<0.001 |
| Engaging in screen-based sedentary behavior on weekdays |  |  |  |  |  |  |
| No | 248 | 38.2% | 239 | 37.8% | NA | NA |
| Yes | 402 | 61.8% | 394 | 62.2% | 1.0 (0.8 to 1.3), p=0.79 | 1.0 (0.7 to 1.3), p=0.82 |
| Engaging in screen-based sedentary behavior on weekend days |  |  |  |  |  |  |
| No | 139 | 21.4% | 102 | 16.1% | NA | NA |
| Yes | 511 | 78.6% | 531 | 83.9% | 0.7 (0.5 to 0.9), p=0.014 | 0.6 (0.4 to 0.8), p=0.001 |
| Engaging in active sports, dance, or games at least once in previous weekend |  |  |  |  |  |  |
| No | 114 | 17.5% | 155 | 24.5% | NA | NA |
| Yes | 536 | 82.5% | 478 | 75.5% | 1.6 (1.2 to 2.1), p=0.003 | 1.5 (1.1 to 2.1), p=0.019 |
| Objectively measured sedentary time (minutes/24 hours) | 539 | 457.2 (99.6) | 531 | 467.2 (93.9) | -15.3 (-38.1 to 7.4), p=0.19 | -8.4 (-31.4 to 14.6), p=0.47 |
| Parent reported sedentary time (minutes/24 hours) | 629 | 204.9 (124.8) | 611 | 216.8 (136.0) | -9.0 (-24.3 to 6.4), p=0.25 | -10.5 (-27.1 to 6.1), p=0.22 |
| Objectively measured MVPA time (minutes/24 hours) | 539 | 65.8 (34.1) | 531 | 63.1 (26.7) | 5.3 (-1.6 to 12.3), p=0.131 | 1.5 (-4.9 to 7.8), p=0.65 |
| Parent reported MVPA time (minutes/24 hours) | 545 | 132.9 [90.0-180.0] | 521 | 124.3 [88.6-184.3] | 3.1 (-8.8 to 15.0), p=0.61 | 3.4 (-9.2 to 16.0), p=0.60 |
| Achieving ≥ 60 minutes MVPA in 24 hours (objectively measured) **^h^** |  |  |  |  |  |  |
| No | 274 | 50.8% | 274 | 51.6% | NA | NA |
| Yes | 265 | 49.2% | 257 | 48.4% | 1.4 (0.8 to 2.4), p=0.26 | 1.2 (0.7 to 2.0), p=0.62 |
| Achieving ≥ 60 minutes MVPA in 24 hours (parent reported) **^h^** |  |  |  |  |  |  |
| No | 46 | 8.0% | 53 | 9.6% | NA | NA |
| Yes | 526 | 92.0% | 500 | 90.4% | 1.1 (0.7 to 1.8), p=0.56 | 0.9 (0.5 to 1.5), p=0.64 |
| **Psycho-social outcomes** | | | | | | |
| PedsQL total score | 650 | 85.8 [77.1- 92.3] | 633 | 83.6 [76.1-91.3] | 1.3 (-0.2 to 2.9), p=0.090 | 1.3 (-0.405 to 3.045), p=0.13 |
| CHU9D utility score | 650 | 0.938 (0.058) | 631 | 0.928 (0.065) | 0.009 (0.001 to 0.016), p=0.020 | 0.008 (-0.001 to 0.017), p=0.085 |
| Kidscreen-52 bullying | 650 | 14 (1.54) | 633 | 13.75 (1.72) | 0.19 (-0.04 to 0.42), p=0.10 | 0.18 (-0.03 to 0.40), p=0.10 |

a: N=the total number of children from whom we collected valid data at the follow up. b: IQR= interquartile range. c: baseline adjusted= adjusted for baseline outcome and school clustering. d: further adjusted=adjusted for baseline outcome, pre-specified school- level (i.e. whether the school provides mid-morning snack, whether the school has an indoor activity room) and child-level socio-demographic (i.e. age, sex and mother education level) and behavioural [daily average servings of fruit and vegetables, weekly servings of unhealthy snacks and sugar added drink, objectively measured time in MVPA (minutes/24 hours) and objectively measured sedentary time (minutes/24 hours)] covariates. e: weight status was defined according WHO growth charts (BMI z score) cut off points. f: based on children who provided valid data. g: adjusted for baseline daily average servings of fruit and vegetables. h: adjusted for baseline objectively measured time in MVPA (per 24 hours).

**Table E: Adjusted differences for the primary and secondary outcomes (anthropometric, diet, physical activity and psychological variables) between control and intervention groups at follow up (in children who were overweight or obese at baseline)**

|  | **Intervention group** | | **Control group** | | **Mean difference or Odds ratio**  **(95% CI), P value** | |
| --- | --- | --- | --- | --- | --- | --- |
|  | **N** ^a^ | **Mean (SD)/** **Median [IQR] ^b^/ %** | **N** ^a^ | **Mean (SD)/ Median [IQR] ^b^/%** | **Baseline adjusted^c^**  **(primary analysis)** | **Further adjusted ^d^**  **(secondary analysis)** |
| **Adiposity related outcomes** | | | | | | |
| BMI z score | 144 | 1.29 (0.97) | 138 | 1.76 (0.91) | -0.49 (-0.73 to -0.25), p<0.001 | -0.56 (-0.82 to -0.30), p<0.001 |
| Obese/Overweight **^e^** |  |  |  |  |  |  |
| No | 44 | 30.6% | 24 | 17.4% | NA | NA |
| Yes | 100 | 69.4% | 114 | 82.6% | 0.2 (0.1 to 0.8), p=0.015 | 0.2 (0.0 to 0.6), p=0.006 |
| Waist circumference | 144 | 67.45 (7.06) | 138 | 68.50 (6.65) | -1.38 (-2.23 to -0.53), p=0.001 | -1.55 (-2.63 to -0.47), p=0.005 |
| Body fat % **^f^** | 78 | 23.5 (6.20) | 81 | 24.43 (5.47) | 0.00 (-0.03 to 0·03), p=0.82 | 0.00 (-0.03 to 0.03), p=0.94 |
| **Blood pressure** | | | | | | |
| Systolic blood pressure (mmHg) | 144 | 106.92 (9.03) | 138 | 107.63 (8.82) | -1.02 (-2.90 to 0.85), p=0.29 | -1.89 (-4.25 to 0.48) , p=0.12 |
| Diastolic blood pressure (mmHg) | 144 | 64.59 (7.88) | 138 | 65.41 (7.82) | -0.86 (-2.96 to 1.24), p=0.42 | -1.15 (-3.55 to 1.24), p=0.35 |
| **Behavioral outcomes** | | | | | | |
| Daily average servings of fruit and vegetables | 117 | 3.0 [2.0-4.0] | 112 | 3.0 [2.0-4.0] | 0.2 (-0.2 to 0.6), p=0.30 | 0.1 (-0.3 to 0.6), p=0.54 |
| ≥ 5 portions of fruit and vegetables **^g^** |  |  |  |  |  |  |
| No | 118 | 81.4% | 128 | 93.4% | NA | NA |
| Yes | 27 | 18.6% | 9 | 6.6% | 3.5 (1.4 to 9.0), p=0.010 | 3.9 (1.1 to 13.8), p=0.038 |
| Weekly average servings of unhealthy snacks and sugar added drinks | 117 | 1.0 [0.0-3.5] | 112 | 2.3 [0.0-3.0] | -0.1 (-1.3 to 1.2), p=0.89 | 0.2 (-1.1 to 1.4), p=0.80 |
| Engaging in screen-based sedentary behavior on weekdays |  |  |  |  |  |  |
| No | 58 | 39.5% | 57 | 41.6% | NA | NA |
| Yes | 89 | 60.5% | 80 | 58.4% | 1.2 (0.7 to 2.0), p=0.62 | 1.2 (0.7 to 2.2), p=0.53 |
| Engaging in screen-based sedentary behavior on weekend days |  |  |  |  |  |  |
| No | 29 | 19.7% | 22 | 16.1% | NA | NA |
| Yes | 118 | 80.3% | 115 | 83.9% | 0.8 (0.4 to 1.6), p=0.55 | 0.9 (0.4 to 1.8), p=0.68 |
| Engaging in active sports, dance, or games at least once in previous weekend |  |  |  |  |  |  |
| No | 28 | 19.0% | 41 | 29.9% | NA | NA |
| Yes | 119 | 81.0% | 96 | 70.1% | 1.7 (1.0 to 3.1), p=0.071 | 1.5 (0.7 to 3.0), p=0.32 |
| Objectively measured sedentary time (minutes/24 hours) | 123 | 483.5 (92.0) | 104 | 472.7 (89.4) | 22.1 (-17.0 to 61.1), p=0.27 | 9.6 (-20.3 to 39.6), p=0.53 |
| Parent reported sedentary time (minutes/24 hours) | 141 | 189.1 (120.7) | 138 | 220.9 (120.7) | -28.0 (-55.2 to -0.9), p=0.043 | -34.4 (-63.8 to -5.04), p=0.022 |
| Objectively measured MVPA time (minutes/24 hours) | 123 | 56.9 (23.8) | 104 | 60.8 (32.3) | -8.9 (-17.9 to 0.0), p=0.050 | -4.0 (-11.7 to 3.6), p=0.30 |
| Parent reported MVPA time (minutes/24 hours) | 117 | 132.9 [90.0-195.0] | 112 | 132.9 [90.0-205.7] | -7.9 (-29.2 to 13.5), p=0.47 | -11.27 (-36.5 to 14.0), p=0.38 |
| Achieving ≥ 60 minutes MVPA in 24 hours (objectively measured) **^h^** |  |  |  |  |  |  |
| No | 73 | 59.4% | 58 | 55.8% | NA | NA |
| Yes | 50 | 40.7% | 46 | 44.2% | 0.5 (0.3 to 1.1), p=0.076 | 0.6 (0.3 to 1.3), p=0.18 |
| Achieving ≥ 60 minutes MVPA in 24 hours (parent reported) **^h^** |  |  |  |  |  |  |
| No | 12 | 9.7% | 5 | 4.3% | NA | NA |
| Yes | 112 | 90.3% | 111 | 95.7% | 0.4 (0.1 to 1.2), p=0.10 | 0.3 (0.1 to 1.0), p=0.048 |
| **Psycho-social outcomes** | | | | | | |
| PedsQL total score | 147 | 84.7 [73.9-93.4] | 137 | 83.6 [73.9-91.3] | 0.8 (-1.7 to 3.3), p=0.53 | 1.1 (-1.9 to 4.3), p=0.46 |
| CHU9D utility score | 147 | 0.930 (0.061) | 137 | 0.927 (0.058) | 0.002 (-0.013 to 0.017), p=0.80 | 0.007 (-0.009 to 0.023), p=0.40 |
| Kidscreen-52 bullying | 147 | 13.72 (1.97) | 137 | 13.73 (2.02) | -0.11 (-0.57 to 0.33), p=0.61 | -0.30 (-0.78 to 0.17), p=0.21 |

a: N=the total number of children from whom we collected valid data at the follow up. b: IQR= interquartile range. c: baseline adjusted= adjusted for baseline outcome and school clustering. d: further adjusted=adjusted for baseline outcome, pre-specified school- level (i.e. whether the school provides mid-morning snack, whether the school has an indoor activity room) and child-level socio-demographic (i.e. age, sex and mother education level) and behavioural [daily average servings of fruit and vegetables, weekly servings of unhealthy snacks and sugar added drink, objectively measured time in MVPA (minutes/24 hours) and objectively measured sedentary time (minutes/24 hours)] covariates. e: weight status was defined according WHO growth charts (BMI z score) cut off points. f: based on children who provided valid data. g: adjusted for baseline daily average servings of fruit and vegetables. h: adjusted for baseline objectively measured time in MVPA (per 24 hours).

**Table F: Adjusted differences for the primary and secondary outcomes (anthropometric, diet, physical activity and psychological variables) between control and intervention groups at follow up - P-values for interaction terms for child baseline weight status**

| **Follow up outcome variable** | **Intervention vs control (BL adjusted)^a^** | **Intervention vs control (further adjusted)^b^** | |
| --- | --- | --- | --- |
|  | p value | p value | |
|  |  |  |  |
| BMI z score |  |  |  |
| Obese/Overweight^c^ | 0.12 | 0.15 |  |
| Waist | <0.001 | <0.001 |  |
| Body fat %^d^ | <0.001 | <0.001 |  |
| Systolic blood pressure (mmHg) | <0.001 | <0.001 |  |
| Diastolic blood pressure (mmHg) | <0.001 | <0.001 |  |
| Daily average servings of fruit and vegetables | 0.19 | 0.31 |  |
| ≥ 5 portions of fruit and vegetables^e^ | 0.98 | 0.89 |  |
| Weekly average servings of unhealthy snacks and sugar added drinks | 0.85 | 0.63 | |
| Objectively measured sedentary time (hours/24 hours) | 0.026 | 0.68 |  |
| Parent reported sedentary time (hours/24 hours) | 0.21 | 0.16 |  |
| Engaging in screen-based sedentary behavior in the weekday | 0.76 | 0.64 |  |
| Engaging in screen-based sedentary behavior in the weekend | 0.65 | 0.51 |  |
| Engaging in active sports, dance, or games for at least once in the last weekend | 0.47 | 0.50 |  |
| Objectively measured MVPA time (minutes/24 hours) | 0.019 | 0.024 |  |
| Parent reported MVPA time (minutes/24 hours) | 0.89 | 0.71 |  |
| Achieving >= 60 minutes MVPA in 24 hours(objectively measured)^f^ | 0.044 | 0.066 |  |
| Achieving >= 60 minutes MVPA in 24 hours(parent reported)^f^ | 0.35 | 0.34 |  |
| PedsQL total score | 0.19 | 0.83 |  |
| CHU9D utility score | 0.14 | 0.84 | |
| Kidscreen-52 bullying | 0.056 | 0.17 | |

BL: baseline. MVPA: moderate - vigorous physical activity. a: adjusted for baseline outcome. b: adjusted for baseline outcome, pre-specified school- level (i.e. whether the school provides mid-morning snack, whether the school has an indoor activity room) and child-level socio-demographic (i.e. age, sex and mother education level) and behavioral [daily average servings of fruit and vegetables, weekly servings of unhealthy snacks and sugar added drink, objectively measured time in MVPA (minutes/24 hours) and objectively measured sedentary time (minutes/24 hours)] covariates. c: adjusted for baseline BMI z-score. d: based on children who were measured by brand new electrodes at 1st follow up. e: adjusted for baseline daily average servings of fruit and vegetables. f: adjusted for baseline objectively measured time in MVPA (per 24 hours).

**Table G: Adjusted differences for the primary and secondary outcomes (anthropometric, diet, physical activity and psychological variables) between control and intervention groups at follow up (in children whose mothers did not have higher education)**

|  | **Intervention group** | | **Control group** | | **Mean difference or Odds ratio**  **(95% CI), P value** | |
| --- | --- | --- | --- | --- | --- | --- |
|  | **N** ^a^ | **Mean (SD)/** **Median [IQR] ^b^/ %** | **N** ^a^ | **Mean (SD)/** **Median [IQR] ^b^ /%** | **Baseline adjusted**  **(primary analysis)** | **Further adjusted ^d^**  **(secondary analysis)** |
| **Adiposity related outcomes** | | | | | | |
| BMI z score | 314 | -0.32 (1.21) | 265 | -0.29 (1.39) | -0.08 (-0.25 to 0.08), p=0.33 | -0.08 (-0.25 to 0.09), p=0.36 |
| Obese/Overweight **^e^** |  |  |  |  |  |  |
| No | 264 | 84.1% | 217 | 81.9% | NA | NA |
| Yes | 50 | 15.9% | 48 | 18.1% | 0.8 (0.3 to 2.1), p=0.66 | 0.9 (0.3 to 2.6), p=0.83 |
| Waist circumference | 315 | 57.50 (6.99) | 265 | 57.66 (7.06) | -0.46 (-1.11 to 0.19), p=0.17 | -0.55 (-1.28 to 0.18), p=0.14 |
| Body fat % **^f^** | 201 | 18.77 (5·66) | 214 | 19.60 (5·07) | -0.01 (-0·03 to 0·01), p=0·29 | -0.01 (-0.03 to 0.01), p=0.24 |
| **Blood pressure** | | | | | | |
| Systolic blood pressure (mmHg) | 315 | 102.23 (8.38) | 265 | 102.02 (8.75) | -0.03(-1.76 to 1.71), p=0.98 | -0.07 (-2.01 to 1.87) , p=0.94 |
| Diastolic blood pressure (mmHg) | 315 | 61.20 (7.13) | 265 | 61.30 (7.38) | -1.06 (-2.72 to 0.59), p=0.21 | -0.73 (-2.31 to 0.85), p=0.37 |
| **Behavioral outcomes** | | | | | | |
| Daily average servings of fruit and vegetables | 256 | 3.0 [2.0-4.0] | 218 | 3.0 [2.0-3.0] | 0.5 (0.3 to 0.7), p<0.001 | 0.5 (0.3 to 0.8), p<0.001 |
| ≥ 5 portions of fruit and vegetables **^g^** |  |  |  |  |  |  |
| No | 251 | 81.5% | 237 | 90.8% | NA | NA |
| Yes | 57 | 18.5% | 24 | 9.2% | 2.3 (1.4 to 4.0), p=0.002 | 2.7 (1.4 to 5.2), p=0.003 |
| Weekly average servings of unhealthy snacks and sugar added drinks | 256 | 1.0 [0.0 -2.9] | 218 | 2.0 [0.0-4.5] | -0.8 (-1.8 to 0.3), p=0.14 | -1.4 (-2.6 to -0.2), p=0.021 |
| Engaging in screen-based sedentary behavior on weekdays |  |  |  |  |  |  |
| No | 101 | 31.7% | 87 | 32.8% | NA | NA |
| Yes | 218 | 68.3% | 178 | 67.2% | 1.1 (0.8 to 1.6), p=0.61 | 1.2 (0.8 to 1.9), p=0.43 |
| Engaging in screen-based sedentary behavior on weekend days |  |  |  |  |  |  |
| No | 61 | 19.1% | 44 | 16.6% | NA | NA |
| Yes | 258 | 80.9% | 221 | 83.4% | 0.8 (0.5 to 1.3), p=0.40 | 0.9 (0.5 to 1.5), p=0.62 |
| Engaging in active sports, dance, or games at least once in previous weekend |  |  |  |  |  |  |
| No | 70 | 21.9% | 79 | 29.8% | NA | NA |
| Yes | 249 | 78.1% | 186 | 70.2% | 1.5 (1.0 to 2.2), p=0.048 | 1.1 (0.7 to 1.7), p=0.78 |
| Objectively measured sedentary time (minutes/24 hours) | 265 | 458.6 (101.2) | 219 | 459.3 (99.6) | -8.9 (-43.8 to 26.0), p=0.62 | -6.2 (-36.7 to 24.4), p=0.69 |
| Parent reported sedentary time (minutes/24 hours) | 305 | 188.2 (114.4) | 257 | 208.2 (136.6) | -20.6 (-40.5 to -0.7), p=0.043 | -24.4 (-48.8 to 0.01), p=0.050 |
| Objectively measured MVPA time (minutes/24 hours) | 265 | 65.0 (29.4) | 219 | 65.3 (29.8) | -0.1 (-9.0 to 8.8), p=0.98 | -0.9 (-8.6 to 6.9), p=0.83 |
| Parent reported MVPA time (minutes/24 hours) | 256 | 137.1 [94.3-200.4] | 218 | 145.7 [99.1-214.3] | -4.3 (-23.5 to 14.9), p=0.66 | -6.1 (-29.9 to 17.7), p=0.62 |
| Achieving ≥ 60 minutes MVPA in 24 hours (objectively measured) **^h^** |  |  |  |  |  |  |
| No | 127 | 47.9% | 110 | 50.2% | NA | NA |
| Yes | 138 | 52.1% | 109 | 49.8% | 1.1 (0.6 to 2.1), p=0.74 | 1.0 (0.5 to 1.9), p>0.99 |
| Achieving ≥ 60 minutes MVPA in 24 hours (parent reported) **^h^** |  |  |  |  |  |  |
| No | 23 | 8.2% | 19 | 8.1% | NA | NA |
| Yes | 257 | 91.8% | 216 | 91.9% | 0.9 (0.5 to 1.7), p=0.74 | 0.6 (0.3 to 1.3), p=0.20 |
| **Psycho-social outcomes** | | | | | | |
| PedsQL total score | 319 | 84.7 [78.2-92.3] | 265 | 84.7 [77.1-91.3] | 1.2 (-0.5 to 3.0), p=0.17 | 0.8 (-1.0 to 2.7), p=0.36 |
| CHU9D utility score | 319 | 0.934 (0.061) | 265 | 0.932 (0.059) | 0.003 (-0.007 to 0.013),p=0.56 | 0.002 (-0.010 to 0.014), p=0.74 |
| Kidscreen-52 bullying | 319 | 14.03 (1.65) | 265 | 13.86 (1.74) | 0.09 (-0.24 to 0.42), p=0.58 | 0.10 (-0.22 to 0.43), p=0.52 |

a: N=the total number of children from whom we collected valid data at the follow up. b: IQR= interquartile range. c: baseline adjusted= adjusted for baseline outcome and school clustering. d: further adjusted=adjusted for baseline outcome, pre-specified school- level (i.e. whether the school provides mid-morning snack, whether the school has an indoor activity room) and child-level socio-demographic (i.e. age, sex and mother education level) and behavioural [daily average servings of fruit and vegetables, weekly servings of unhealthy snacks and sugar added drink, objectively measured time in MVPA (minutes/24 hours) and objectively measured sedentary time (minutes/24 hours)] covariates. e: weight status was defined according WHO growth charts (BMI z score) cut off points. f: based on children who provided valid data. g: adjusted for baseline daily average servings of fruit and vegetables. h: adjusted for baseline objectively measured time in MVPA (per 24 hours).

**Table H: Adjusted differences for the primary and secondary outcomes (anthropometric, diet, physical activity and psychological variables) between control and intervention groups at follow up (in children whose mothers had higher education)**

|  | **Intervention group** | | **Control group** | | **Mean difference or Odds ratio**  **(95% CI), P value** | |
| --- | --- | --- | --- | --- | --- | --- |
|  | **N** ^a^ | **Mean (SD)/** **Median [IQR] ^b^/ %** | **N** ^a^ | **Mean (SD)/** **Median [IQR] ^b^/%** | **Baseline adjusted^c^**  **(primary analysis)** | **Further adjusted ^d^**  **(secondary analysis)** |
| **Adiposity related outcomes** | | | | | | |
| BMI z score | 473 | -0.36 (1.22) | 473 | -0.18 (1.33) | -0.18 (-0.31 to 0.08), p=0.005 | -0.08 (-0.25 to -0.06), p=0.003 |
| Obese/Overweight **^e^** |  |  |  |  |  |  |
| No | 400 | 84.6% | 381 | 80.5% | NA | NA |
| Yes | 73 | 15.4% | 92 | 19.5% | 0.3 (0.1 to 0.7), p=0.006 | 0.4 (0.2 to 0.9), p=0.032 |
| Waist circumference | 473 | 57.51 (6.65) | 477 | 58.02 (6.82) | -0.38 (-0.98 to 0.21), p=0.21 | -0.60 (-1.18 to - 0.02), p=0.042 |
| Body fat % **^f^** | 267 | 19.13 (5·64) | 194 | 20.59 (6.12) | -0.02 (-0·04 to 0·00), p=0·058 | -0.01 (-0.03 to 0.00), p=0.068 |
| **Blood pressure** | | | | | | |
| Systolic blood pressure (mmHg) | 473 | 101.08 (8.71) | 477 | 101.09 (9.06) | -0.22(-1.71 to 1.26), p=0.77 | -0.50 (-1.95 to 0.95) , p=0.50 |
| Diastolic blood pressure (mmHg) | 473 | 60.60 (7.13) | 477 | 61.21 (7.03) | -0.66 (-2.180to 0.86), p=0.39 | -0.80 (-2.31 to 0.72), p=0.30 |
| **Behavioral outcomes** | | | | | | |
| Daily average servings of fruit and vegetables | 401 | 3.0 [2.0-4.0] | 400 | 3.0 [2.0-4.0] | 0.2 (0.0 to 0.5), p=0.048 | 0.2 (0.0 to 0.5), p=0.038 |
| ≥ 5 portions of fruit and vegetables **^g^** |  |  |  |  |  |  |
| No | 392 | 83.2% | 428 | 90.1% | NA | NA |
| Yes | 79 | 16.8% | 47 | 9.9% | 1.8 (1.2 to 2.7), p=0.004 | 1.9 (1.2 to 3.0), p=0.005 |
| Weekly average servings of unhealthy snacks and sugar added drinks | 401 | 1.0 [0.0-3.0] | 400 | 2.5 [0.0-3.5] | -0.9 (-1.7 to -0.1), p=0.031 | -0.42 (-1.0 to 0.11), p=0.12 |
| Engaging in screen-based sedentary behavior on weekdays |  |  |  |  |  |  |
| No | 205 | 43.5% | 200 | 41.8% | NA | NA |
| Yes | 266 | 56.5% | 279 | 58.2% | 1.0 (0.7 to 1.3), p=0.78 | 0.9 (0.7 to 1.3), p=0.56 |
| Engaging in screen-based sedentary behavior on weekend days |  |  |  |  |  |  |
| No | 107 | 22.7% | 71 | 14.8% | NA | NA |
| Yes | 364 | 77.3% | 408 | 85.2% | 0.6 (0.4 to 0.8), p=0.002 | 0.5 (0.3 to 0.7), p<0.001 |
| Engaging in active sports, dance, or games at least once in previous weekend |  |  |  |  |  |  |
| No | 70 | 14.9% | 108 | 22.5% | NA | NA |
| Yes | 401 | 85.1% | 371 | 77.5% | 1.7 (1.2 to 2.4), p=0.002 | 1.8 (1.2 to 2.6), p=0.003 |
| Objectively measured sedentary time (minutes/24 hours) | 92 | 463.2 (96.9) | 395 | 476.1 (85.3) | -12.6 (-32.8 to 7.5), p=0.22 | -9.6 (-29.0 to 9.8), p=0.33 |
| Parent reported sedentary time (minutes/24 hours) | 459 | 212.1 (129.8) | 473 | 221.9 (131.6) | -6.7 (-23.5 to 10.0), p=0.44 | -8.9 (-26.9 to 9.0), p=0.33 |
| Objectively measured MVPA time (minutes/24 hours) | 392 | 63.9 (34.7) | 395 | 61.0 (26.3) | 5.3 (-2.0 to 12.6), p=0.15 | 1.2 (-5.4 to 7.8), p=0.72 |
| Parent reported MVPA time (minutes/24 hours) | 401 | 128.6 [88.6-175.7] | 400 | 120 [85.7-172.5] | 1.9 (-11.5 to 15.2), p=0.79 | 3.9 (-9.3 to 17.1), p=0.56 |
| Achieving ≥ 60 minutes MVPA in 24 hours (objectively measured) **^h^** |  |  |  |  |  |  |
| No | 216 | 55.1% | 213 | 53.9% | NA | NA |
| Yes | 176 | 44.9% | 182 | 46.1% | 1.2 (0.7 to 2.2), p=0.44 | 1.0 (0.6 to 1.9), p=0.96 |
| Achieving ≥ 60 minutes MVPA in 24 hours (parent reported) **^h^** |  |  |  |  |  |  |
| No | 34 | 8.3% | 36 | 8.6% | NA | NA |
| Yes | 377 | 91.7% | 383 | 91.4% | 1.0 (0.6 to 1.8), p=0.96 | 0.9 (0.5 to 1.6), p=0.75 |
| **Psycho-social outcomes** | | | | | | |
| PedsQL total score | 471 | 85.8 [76.1-92.3] | 479 | 83.6 [75.0-91.3] | 1.2 (-0.5 to 2.9), p=0.13 | 1.3 (-0.6 to 3.1), p=0.19 |
| CHU9D utility score | 471 | 0.938 (0.057) | 477 | 0.927 (0.067) | 0.009 (-0.000 to 0.019), p=0.057 | 0.010 (0.000 to 0.021), p=0.040 |
| Kidscreen-52 bullying | 471 | 13.88 (1.62) | 479 | 13.69 (1.81) | 0.12 (-0.13 to 0.37), p=0.34 | 0.05 (-0.23 to 0.33), p=0.72 |

a: N=the total number of children from whom we collected valid data at the follow up. b: IQR= interquartile range. c: baseline adjusted= adjusted for baseline outcome and school clustering. d: further adjusted=adjusted for baseline outcome, pre-specified school- level (i.e. whether the school provides mid-morning snack, whether the school has an indoor activity room) and child-level socio-demographic (i.e. age, sex and mother education level) and behavioural [daily average servings of fruit and vegetables, weekly servings of unhealthy snacks and sugar added drink, objectively measured time in MVPA (minutes/24 hours) and objectively measured sedentary time (minutes/24 hours)] covariates. e: weight status was defined according WHO growth charts (BMI z score) cut off points. f: based on children who provided valid data. g: adjusted for baseline daily average servings of fruit and vegetables. h: adjusted for baseline objectively measured time in MVPA (per 24 hours).

**Table I: Adjusted differences for the primary and secondary outcomes (anthropometric, diet, physical activity and psychological variables) between control and intervention groups at follow up - P-values for interaction terms for mother education level**

| **Follow up outcome variable** | **Intervention vs control (BL adjusted)^a^** | **Intervention vs control (further adjusted)^b^** | |
| --- | --- | --- | --- |
|  | p value | p value | |
|  |  |  |  |
| BMI z score | 0.76 | 0.78 |  |
| Obese/Overweight^c^ | 0.16 | 0.27 |  |
| Waist | 0.83 | 0.54 |  |
| Body fat %^d^ | 0.98 | 0.46 |  |
| Systolic blood pressure (mmHg) | 0.57 | 0.32 |  |
| Diastolic blood pressure (mmHg) | 0.95 | 0.65 |  |
| Daily average servings of fruit and vegetables | 0.71 | 0.62 |  |
| ≥ 5 portions of fruit and vegetables^e^ | 0.25 | 0.35 |  |
| Weekly average servings of unhealthy snacks and sugar added drinks | 0.66 | 0.91 |  |
| Objectively measured sedentary time (hours/24 hours) | 0.32 | 0.57 |  |
| Parent reported sedentary time (hours/24 hours) | 0.08 | 0.18 |  |
| Engaging in screen-based sedentary behavior in the weekday | 0.004 | 0.013 |  |
| Engaging in screen-based sedentary behavior in the weekend | 0.35 | 0.16 |  |
| Engaging in active sports, dance, or games for at least once in the last weekend | 0.008 | 0.002 |  |
| Objectively measured MVPA time (minutes/24 hours) | 0.97 | 0.59 |  |
| Parent reported MVPA time (minutes/24 hours) | 0.004 | 0.012 |  |
| Achieving >= 60 minutes MVPA in 24 hours(objectively measured)^f^ | 0.47 | 0.70 |  |
| Achieving >= 60 minutes MVPA in 24 hours(parent reported)^f^ | 0.81 | 0.82 |  |
| PedsQL total score | 0.92 | 0.91 |  |
| CHU9D utility score | 0.51 | 0.29 | |
| Kidscreen-52 bullying | 0.41 | 0.022 | |

BL: baseline. MVPA: moderate - vigorous physical activity. a: adjusted for baseline outcome. b: adjusted for baseline outcome, pre-specified school- level (i.e. whether the school provides mid-morning snack, whether the school has an indoor activity room) and child-level socio-demographic (i.e. age, sex and mother education level) and behavioral [daily average servings of fruit and vegetables, weekly servings of unhealthy snacks and sugar added drink, objectively measured time in MVPA (minutes/24 hours) and objectively measured sedentary time (minutes/24 hours)] covariates. c: adjusted for baseline BMI z-score. d: based on children who were measured by brand new electrodes at 1st follow up. e: adjusted for baseline daily average servings of fruit and vegetables. f: adjusted for baseline objectively measured time in MVPA (per 24 hours).
