# Supplementary material for: The CHIRPY DRAGON intervention in preventing obesity in Chinese primary-school--aged children: A cluster-randomised controlled trial
Source: PLoS Med. 2019 Nov 26;16(11):e1002971. doi: 10.1371/journal.pmed.1002971 (PMC6879117; doi:10.1371/journal.pmed.1002971)
Supplement: S1 Table — CHIRPY DRAGON, Chinese Primary School Children Physical Activity and Dietary Behaviour Changes Intervention (DOCX) [file pmed.1002971.s003.docx]

**S1 Table: Summary of the CHIRPY DRAGON intervention components and delivery**

| **Target Audience** | **Activities** | **Number of Sessions and Setting** |
| --- | --- | --- |
| **Component 1: To improve childhood obesity related knowledge, skills and behaviours among children and their main carers** | | |
| Main carers (parents/guardians and grandparents) | (A) Interactive education workshops for carers with summarising leaflet to take home (focused on correcting common misperceptions identified through our formative research in relation to child healthy weight and healthy behaviours, and introducing practical parenting tips for encouraging healthy behavioural change in children) | 2, school-based |
| Children | (B) Interactive educational activities for children (focused on key messages related to healthy  eating and an active lifestyle) | 4, school-based |
| Children and their main carers | (C) Child setting challenging but achievable healthy behaviour goals & child self-monitoring, with parent and CHIRPY DRAGON teacher feedback and prize for highest achievers *(see next page for examples and explanation of child self-monitoring fun cards)* | Home based. Predetermined daily themes (eating at least five portions of fruit and vegetables daily; engaging in no more than 2 hours of sedentary screen-based activities a day; and consuming fewer snacks and drinks that are high in sugar and/or fats) were rotated every 2 weeks, plus one continuous weekend challenge (see Component 3, part B) |
|  | (D) Health knowledge Quiz | 1, school-based |
| **Component 2: To improve the nutritional quality of school lunch provision (usually a set lunch box for each child)** | | |
| School lunch providers and catering staff | (A) Introduce school lunch improvement goals that were agreed jointly by researchers and school lunch providers and then tested by school lunch providers (including both commercial suppliers and school funded catering units) | 1 introduction meeting held in the Guangzhou CDC |
|  | (B) Supportive evaluation of school lunch provision against agreed goals and feedback | Continued throughout the intervention year, school-based |
| **Component 3: To increase children’s physical activity level outside school** | | |
| Children and their parents | (A) Taster session to teach fun & active family games that could be undertaken with minimal equipment and space at home | 2, school-based |
|  | (B) Assign homework (a family-wide healthy behaviour challenge) – practicing taught family games or other non-sedentary activities involving the child and parents for at least 30 minutes every weekend | Home based. Continuous weekend challenge |
| **Component 4: To increase children’s physical activity level within school** | | |
| Children and school staff | (A) Situation analysis in relation to current implementation of the Chinese national standard of having one-hour physical activity on campus every school day | Monthly meetings held throughout the intervention year, school-based |
|  | (B) Setting monthly goals (measurable and achievable) and action plans to meet, maintain or exceed the national standard, with continuous evaluation and feedback |  |

**Examples and explanation of child self-monitoring cards**

Below are two examples of child self-monitoring cards, with translation and detailed description.


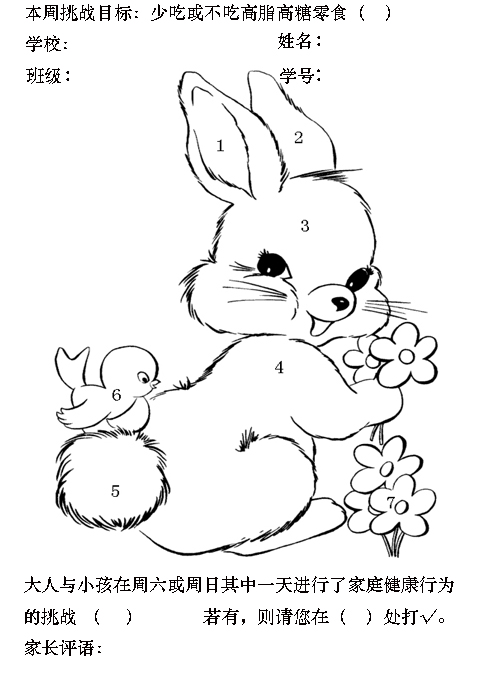

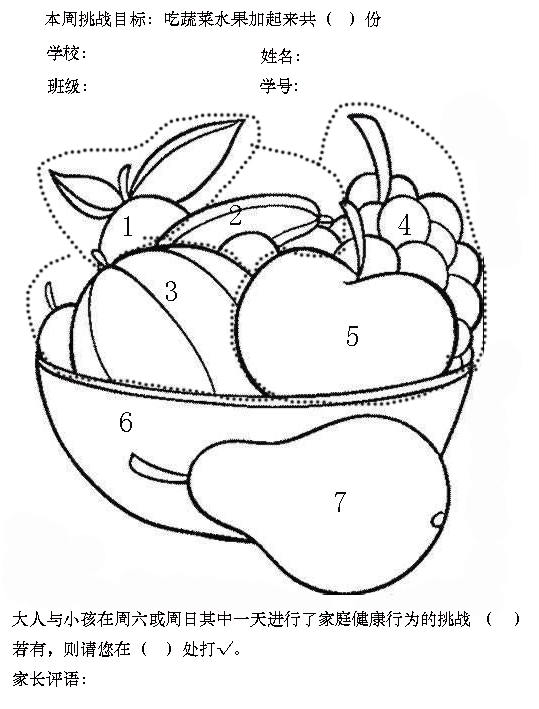


Above are examples of the self-monitoring cards that children were asked to fill in, with a translated version below:

‘The challenge for this week…………..

School name: Child’s name:

Class number: Class ID:

‘The challenge for this week…………..

School name: Child’s name:

Class number: Class ID:

*………………..*


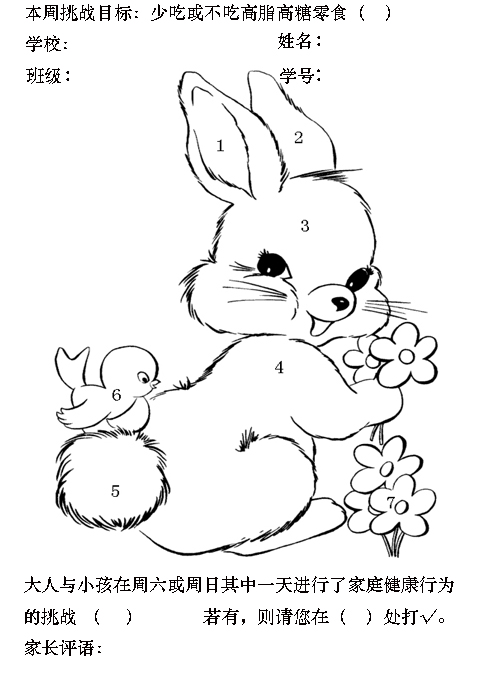

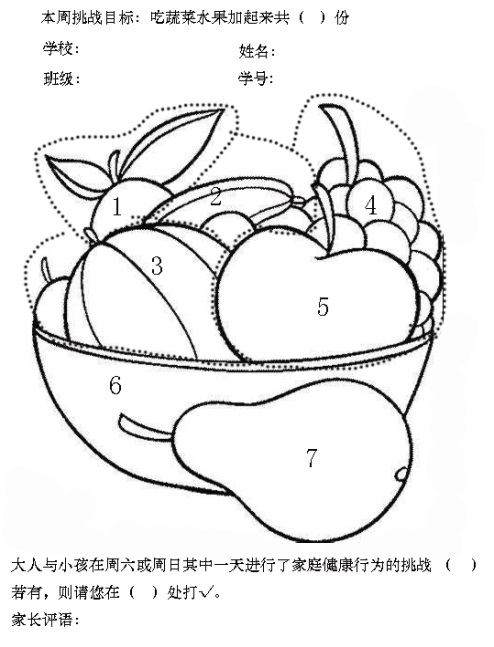


*Has the child and his/her family engaged in active games or activities for at least 30 minutes on Saturday/Sunday (🗸Tick if yes ):*

*Comments from parents or guardians for this week:*

*Has the child and his/her family engaged in active games or activities for at least 30 minutes on Saturday/Sunday (🗸Tick if yes ):*

*Comments from parents or guardians for this week:*

The cartoon image was updated every week to reflect the specific behavioural challenge for the week and to maintain children’s interest. The cartoon images have 7 parts, each representing one day of the week. Children were asked to colour in one part each day, if they had met their behavioural target for that day. Thus a fully coloured in image indicated the child had met his/her behavioural targets on all the days of that week. Under each image there was an allocated space for parents/guardians to provide written feedback on the child’s and family’s behaviour/progress in relation to 1) the weekend only behavioural challenge, and 2) both the weekend and week day challenges of the week.

**Summary of staff training and quality control programme**

Group training (provided by Bai Li, first author) was delivered in the format of an interactive workshop over one week. Topics covered included:

1) background epidemiology and the importance of childhood obesity prevention;

2) aims of the study and details of the intervention

3) theory and practical implementation of a cluster randomised controlled trial (with a particular focus on strategies to avoid potential contamination)

4) how to effectively engage with children, school leaders, teachers and family members

5) project timeframe and the role of each of the project team members (responsibilities and reporting lines)

6) how to report and handle problems

All CHIRPY DRAGON teachers completed and passed a face-to-face, oral assessment of their understanding and communication skills required to coordinate the intervention in their assigned schools.

In addition to group training, each of the CHIRPY DRAGON teachers practiced delivery and role play of at least one ‘mock workshop’ for each of the planned intervention workshops. During these role-plays, they also practiced and received feedback on how to handle various unexpected situations (e.g. challenging questions or behaviours from the audience) that could occur in real intervention workshops.

We sought to standardise delivery through a variety of means including a structured training programme for project staff (summarised above) and development of a detailed training manual in Chinese. In addition, school staff received handbooks (two different versions: one for school principals, the other for class teachers) which detailed key project information such as the intervention timetable (tailored to each school and class), planned activities and the responsibilities of different staff. Finally, we had a quality control process in place for the CHIRPY DRAGON teachers, whereby they reported activities weekly to Bai Li (first author). This included discussion of any intervention protocol deviations, problems in delivery or participant attendance, queries from participants or schools or any other concerns. This allowed problems to be tackled in a timely way and solutions and learning, with any advice to be shared more widely among local project team members.
