# Supplementary material for: The CHIRPY DRAGON intervention in preventing obesity in Chinese primary-school--aged children: A cluster-randomised controlled trial
Source: PLoS Med. 2019 Nov 26;16(11):e1002971. doi: 10.1371/journal.pmed.1002971 (PMC6879117; doi:10.1371/journal.pmed.1002971)
Supplement: S2 Table — (DOCX) [file pmed.1002971.s004.docx]

**S2 Table: Summary of data collection methods**

| **Type of measurement** | **Instrument** | **Number of measures** | **Method of measurement** |
| --- | --- | --- | --- |
| **Child anthropometric data** | | | |
| **Weight** | An electronic scale (JH-1993T, weighting Apparatus Co. Ltd. Dalian) | Once, at all time points | Barefoot and in light clothing |
| **Birth weight** | Purposely developed question tested in the feasibility study | Once, at all time points | Parent questionnaire |
| **Body fat %** | Single-frequency ImpediMed machine (ImpDF50; Impedimed Pty Ltd, Australia). | Once, at all time points | Barefoot and in light clothing, all metal accessories removed from the body |
| **Blood pressure** | Automated monitors (Omron HEM-7211, Dalian) | Twice (third measure if error reading, or if one value outside  normal range), at all time points | 3 minutes seated-rest before and between readings |
| **Height (to nearest 0.1 cm)** | TGZ type height tester (Dalian) | Twice (third measure if difference >0.4cm) | Barefoot and in light clothing |
| **Waist circumference (to nearest 0.1 cm)** | Flexible, non-stretch, cloth tape measure |  | Measured midway between the rib cage and the iliac crest |
| **Other child-level data** | | | |
| **Consumption frequency of common food and drinks** (weekly average servings of unhealthy snacks and sugar added drinks can be derived) | Adapted Leeds FFQ | Once, at all time points | Parent questionnaire |
| **Daily average servings of fruit and vegetables** | Leeds FFQ | Once, at all time points | Parent questionnaire |
| **≥ 5 portions of fruit and veg daily, n (%)** | Leeds FFQ | Once, at all time points | Parent questionnaire |
| **Objective measure of physical activity/sedentary behaviour** [MVPA time(minutes/24 hours) and sedentary time (hours/24hours) can be derived] | Wrist worn accelerometer (GENEActiv Original, Activinsights Ltd, Cambridge) | Once (worn continuously for 5 days, including a weekend), at all time points | Fitted in school by trained researcher on the wrist of the non-dominant hand |
| **Subjective measure of physical activity** | Adapting the Godin Leisure Time Exercise Questionnaire | Once, at all time points | Parent questionnaire |
| **Frequency and types of physical activities inside and outside school** (whether the child engaged in active sports/dancing/games for at least once in the last weekend can be derived) | PAQ-C | Once, at all time points | Child questionnaire (researcher administered) |
| **Time spent in screen-based sedentary behaviour in weekdays and in the weekend** | Purposely developed questions tested in the feasibility study | Once, at all time points | Child questionnaire (researcher administered) |
| **Quality of life (child)** | Paediatric Quality of Life Inventory (PedsQL) 8-12 years (validated Chinese version) | Once, at all time points | Child questionnaire (researcher administered) |
| **Social acceptance** | Translated Kidscreen-52 health questionnaire for children and young people |  |  |
| **Socio-demographic data** | | | |
| **Date of birth, sex and mother education level** | Purposely developed questions tested in the feasibility study | once, at all time points | Parent questionnaire and school record on child birth date and sex |
