# Supplementary material for: The CHIRPY DRAGON intervention in preventing obesity in Chinese primary-school--aged children: A cluster-randomised controlled trial
Source: PLoS Med. 2019 Nov 26;16(11):e1002971. doi: 10.1371/journal.pmed.1002971 (PMC6879117; doi:10.1371/journal.pmed.1002971)
Supplement: S3 Table — (DOCX) [file pmed.1002971.s005.docx]

**S3 Table: Comparison of trial completers (n=1586) and those lost to follow up (n=55) by baseline characteristics**

|  | **Trial completers**  (n=1,586) | **Lost to follow up**  (n=55) |
| --- | --- | --- |
| **Sex** | | |
| Boys | 857 (54.0%) | 37 (67.3%) |
| Girls | 729 (46.0%) | 18 (32.7%) |
| **Mother education level** | | |
| Lower education | | |
| None | 2 (0.1%) | 0 (0.0%) |
| School education (Primary and Middle schools) | 295 (19.3%) | 9 (16.7%) |
| Occupation college | 283 (18.5%) | 9 (16.7%) |
| Higher education | | |
| University education (Undergraduate level) | 836 (54.6%) | 31 (57.3%) |
| Postgraduate education | 114 (7.5%) | 5 (9.3%) |
| Not known | 56 | 1 |
| **Baseline weight status*** |  |  |
| Non overweight | 1281 (81.9%) | 47 (85.4%) |
| Overweight/obese | 282 (18.1%) | 8 (14.6%) |
| Not known~ | 23 | 0 |

Data are n (%); *based on WHO 2007 Growth Chart; ~ absent at baseline assessment date or recorded data was classified as invalid.
